# Phosphorylation of Tet3 by cdk5 is critical for robust activation of *BRN2* during neuronal differentiation

**DOI:** 10.1093/nar/gkz1144

**Published:** 2019-12-06

**Authors:** Vinay Kumar Rao, Adusumalli Swarnaseetha, Guo-Hong Tham, Wei-Qi Lin, Bin-Bin Han, Touati Benoukraf, Guo-Liang Xu, Chin-Tong Ong

**Affiliations:** 1 Temasek Life Sciences Laboratory, National University of Singapore, Singapore 117604, Singapore; 2 Department of Biological Sciences, National University of Singapore, Singapore 117558, Singapore; 3 State Key Laboratory of Molecular Biology, CAS Center for Excellence in Molecular Cell Science, Shanghai Institute of Biochemistry and Cell Biology, Chinese Academy of Sciences, Shanghai 200031, China; 4 Cancer Science Institute of Singapore, National University of Singapore, Singapore 117599, Singapore; 5 Discipline of Genetics, Faculty of Medicine, Memorial University of Newfoundland, St. John's, NL A1B 3V6, Canada

## Abstract

Tet3 regulates the dynamic balance between 5-methylcyotsine (5mC) and 5-hydroxymethylcytosine (5hmC) in DNA during brain development and homeostasis. However, it remains unclear how its functions are modulated in a context-dependent manner during neuronal differentiation. Here, we show that cyclin-dependent kinase 5 (cdk5) phosphorylates Tet3 at the highly conserved serine 1310 and 1379 residues within its catalytic domain, changing its *in vitro* dioxygenase activity. Interestingly, when stably expressed in *Tet1, 2, 3* triple-knockout mouse embryonic stem cells (ESCs), wild-type Tet3 induces higher level of 5hmC and concomitant expression of genes associated with neurogenesis whereas phosphor-mutant (S1310A/S1379A) Tet3 causes elevated 5hmC and expression of genes that are linked to metabolic processes. Consistent with this observation, *Tet3*-knockout mouse ESCs rescued with wild-type Tet3 have higher level of 5hmC at the promoter of neuron-specific gene *BRN2* when compared to cells that expressed phosphor-mutant Tet3. Wild-type and phosphor-mutant Tet3 also exhibit differential binding affinity to histone variant H2A.Z. The differential 5hmC enrichment and H2A.Z occupancy at *BRN2* promoter is correlated with higher gene expression and more efficient neuronal differentiation of ESCs that expressed wild-type Tet3. Taken together, our results suggest that cdk5-mediated phosphorylation of Tet3 is required for robust activation of neuronal differentiation program.

## INTRODUCTION

DNA methylation at cytosine is a highly dynamic epigenetic modification that is crucial for gene regulation during cellular differentiation and homeostasis (1). Tet1, 2 and 3 enzymes belong to a family of DNA dioxygenases that oxidize 5-methylcytosine (5mC) by using α-ketoglutarate and Fe (II) ions as co-factors (2,3). Iterative oxidation by Tet enzymes generates 5-hydroxymethylcytosine (5hmC), 5-formylcytosine (5fC) and 5-carboxylcytosine (5caC), which are eventually converted to cytosine through the thymine DNA glycosylase-dependent (TDG) base-excision repair (BER) pathway (4,5). While localization of 5mC at the promoter is frequently associated with gene repression, the presence of 5hmC at the promoter-distal regulatory elements and intragenic regions is highly linked to transcriptional activation in mouse ESCs (6–12) and during cellular differentiation (13–16). Interestingly, insulator protein CTCF has strong binding affinity for 5hmC (17) with its DNA binding motif highly enriched in 5hmC regions (6,8,12,18). As CTCF plays an important role in nuclear organization (19), it is plausible that changes to the genomic distribution of 5hmC may inevitably affect CTCF-mediated long-range chromosomal interactions.

Tet enzymes regulate different aspects of neuronal development and homeostasis, correspondingly, the 5hmC level is found to be highest in the brain compared to other tissues (20–22). During neuronal differentiation of mouse ESCs, Tet3 biases neuro-ectodermal over mesodermal specification by inhibiting Wnt signaling (23). The presence of Tet3 in neuronal progenitor cells (NPCs) is also required to prevent apoptosis and promote terminal differentiation into neurons (24). In the presence of Forskolin, Tet3 can induce fibroblast to form functional neurons by regulating the 5hmC state at neuron-specific gene clusters (25). In addition, Tet3 has also been shown to regulate the expression of *GluR1* in response to synaptic activity (26) and *gephyrin* gene during rapid behavioural adaptation (27). It is required for the activation of axon guidance and ion channel genes in granule cells during cerebellar circuit formation (28). In adult brain, Tet3 can prevent neurodegeneration (29) and inhibit the renewal of glioblastoma stem cells (30). While the functions of Tet3 has been shown to be regulated by O-GlcNAc transferase (31–33), PCG7 (34) and microRNA-15b (35), it remains unclear if other post-translational modifications may contribute to its diverse regulatory roles in the brain tissues.

In this study, we identified multiple novel phosphorylation sites in human Tet3 protein through mass spectrometry analysis. Interestingly, serine 1310 and 1379 within human Tet3 catalytic domain contain the highly conserved SPx(K/R) motif. These serine residues are found to be phosphorylated by cyclin-dependent kinase 5 (cdk5), an atypical cdk that regulates brain development and maintain proper neurological functions (36,37). Stable expression of either wild-type or phosphor-mutant (S1310A/S1379A) Tet3 in *Tet1, 2*, *3* triple-knockout mouse ESCs lead to differential gene expression patterns. Wild-type Tet3 induces higher levels of 5hmC and concomitant expression of genes that are associated with neurogenesis. On the other hand, phosphor-mutant Tet3 elicited higher 5hmC and expression of genes that are involved in metabolic processes. Consistent with the overlapping roles of Tet3 and cdk5 in neuronal development, wild-type but not phosphor-mutant Tet3 promotes efficient neuronal differentiation of mouse ESCs by increasing the expression of neuron-specific transcription factor BRN2. Mechanistically, this was achieved through differential 5hmC enrichment and H2A.Z occupancy at the promoter of *BRN2* gene. Taken together, our data suggest that cdk5-mediated phosphorylation of Tet3 is required for the robust activation of neuronal genes during differentiation.

## MATERIALS AND METHODS

### Molecular cloning

Flag-tagged mouse Tet1 and Tet3 catalytic domains (Tet1CD, Tet3CD), Flag-tagged full length human Tet3 (SBP-N-terminal-Flag-hTet3-IRES-GFP) and lentiviral vector (pLV-EF1α-N-terminal-Flag-IRES-puro) were kind gifts from Xiaodong Cheng, Xiaochun Yu and Yih-Cherng Liou respectively. Site-directed mutagenesis was carried out as described previously (38) to generate wild-type and phosphor-mutant Tet3. Mouse Tet3 catalytic domain was cloned into pGEX-4T-1 GST vector by EcoRI/NotI double digestion while full-length human Tet3 cDNA was introduced into the lentiviral vector by XbaI/PspXI restriction sites. Primers sequences and their standard curves are summarized in Supplementary Tables S8 and S9 respectively.

### *In vitro* kinase assay

Phosphorylation was carried out in a 25 μl reaction buffer containing 4.5 μg of purified GST-Tet3CD or Flag-Tet3CD proteins, 1× NEB kinase buffer, 1 mM ATP in the presence or absence of 1 μl Cdk5/p25 (Enzo Life Sciences) for 1 hr at 30°C. 8 μl of *in vitro* kinase reaction was boiled in Laemmli SDS buffer and subjected to western analysis.

### *In vitro* dioxygenase activity

The dioxygenase reaction was carried out in a 40 μl buffer (50 mM pH 8.0 HEPES, 100 mM NaCl, 50 μM Fe^2+^, 2 mM ascorbic acid, 1 mM α-ketoglutarate) containing 400 ng of double-stranded 5mC DNA fragments and 8 μl of the *in vitro* kinase reaction described above (equivalent to ∼1.5 μg Tet3) for 40 min at 37°C. The reaction was stopped by the addition of 20 μg proteinase K and 30 min incubation at room temperature. The modified DNA fragments were resolved on a 1% agarose gel and transferred to Zeta-probe membrane (Bio-rad) by Southern blot according to manufacturer protocol. The transferred membrane was UV-crosslinked and probed with anti-5hmC antibody (Active motif) according to standard Western protocol.

### Immunoprecipitation (IP)

HEK 293 cells harvested 48 hr post-transfection was washed once with ice-cold PBS. All IP steps were carried out at 4°C. HEK293 cell pellets were lysed in ice-cold RIPA buffer [10 mM Tris pH 8.0, 1 mM EDTA, 0.5 mM EGTA, 1% triton, 0.1% sodium deoxycholate, 0.1% SDS, 140 mM NaCl, 0.5 mM DTT, 1× cOmplete Mini EDTA-free protease inhibitor (Roche) and phosphatase inhibitor cocktail (P5726 and P0044, Sigma)] for 20 min with agitation. Lysates were clarified by centrifugation at 16 000 g for 10 min and protein concentrations were determined by Bradford assays (Bio-Rad). For mass spectrometry analysis, lysate was incubated 2 h with pre-washed anti-Flag M2 affinity gel (Sigma). For western analysis, lysate was incubated for 1 h with protein A/G Sepharose (GE healthcare) pre-bound to anti-Flag antibody (Sigma). The IP complexes were washed three times with RIPA buffer, boiled in Laemmli SDS buffer (with 100mM DTT) for 5 min and resolved by SDS-PAGE.

To determine interaction between Flag-tagged Tet3 with H2A.Z in mouse ESCs, cell pellets were lysed in ice-cold TBST buffer [50 mM Tris–HCl, pH 7.4, 150 mM NaCl, 1 mM EDTA, and 1% Triton X-100 1× cOmplete Mini EDTA-free protease inhibitor (Roche)] for 20 min with agitation. IP was performed overnight using mouse IgG agarose (Sigma) or anti-Flag M2 affinity gel and washed four times with TBST buffer.

### Generation of phosphor-Tet3 (pTet3) antibody

The pTet3 antibody was generated by Genscript Antibody Group using the 16 amino acids peptide C-GQYSGGPSM[pSER]PKRTN, where C is the conjugated cysteine (Supplementary Figure S1A).

### Cell culture

HEK 293 cells were maintained in Dulbecco's modified Eagle's medium (DMEM) GlutaMAX™ (Gibco) supplemented with 10% FBS, 0.1 mM nonessential amino acids, 1.0 mM sodium pyruvate, and 100 units/ml penicillin/streptomycin.

*Tet1*, *2* and *3* triple knockout (TKO) and *Tet3* KO mouse ESCs of mixed *C57BL/6J-129V* background (39) were cultured in 2i medium composed of DMEM GlutaMAX^TM^ (Gibco), 15% heat-inactivated ES-qualified FBS (Gibco), 0.1 mM nonessential amino acids, 1.0 mM sodium pyruvate, 1 μM PD0325901 (StemGent), 3 μM CHIR99021 (Axon Medchem), 1000 U/ml murine leukemia inhibitory factor (LIF, Chemicon) and 0.1 mM β-mercaptoethanol. Mouse ESCs were passaged using StemPro Accutase (Gibco) and seeded on feeder-free flask pre-coated with attachment factor (Gibco).

Neuronal differentiation was performed according to published protocol (Figure 5B) (40). Briefly, 4 million mouse ESCs were cultured in 15 ml of cell aggregate (CA) medium (DMEM GlutaMAX™, 10% FBS, 0.1 mM nonessential amino acids, 1.0 mM sodium pyruvate and 0.1 mM β-mercaptoethanol) in a 100 mm bacteriological Petri dish (Greiner) for 4 days. Cellular aggregates that resembled embryoid bodies (EB) were cultured for 4 additional days in CA medium containing 5 μM retinoic acid (RA) to form neuronal progenitor cells (NPCs). On day 8, NPCs were segregated by trypsin, resuspended in N2 medium, counted and seeded equally (for wild-type & phosphor-mutant Tet3 lines) at a density of 0.5e6/well in 12-well tissue culture plate pre-coated with PORN (Sigma) and laminin (Roche). After 48 h of culture in N2 medium, the neurons were replenished with B27 complete medium (both Gibco).

### Mice

*C57BL/6J* mice were housed and their brain tissues were harvested according to the guidelines set by the Temasek Life Sciences Laboratory IACUC.

### Transfection and lentiviral transduction

Plasmid DNA and siRNA molecules were transiently transfected into HEK293 cells using Lipofectamine^®^ 2000 reagent (Invitrogen) according to manufacturer protocol. For drug treatment, DMSO or 20 μM of roscovitine (Cell Signaling Technology) was added to HEK293 cells 24 h after transfection and incubated for additional 24 h. For dot blot assay in Figure 2E to G, cells transfected with SBP-Flag-hTet3-(Wt/AA)-IRES-GFP constructs for 48 h were FACS-sorted (BD FACSVantage SE) based on their GFP signal.

Lenti-X lentiviral expression system (Clontech) was used to generate stable mouse ESC lines that expressed either empty lentiviral plasmid (Em), wild-type (Wt), phosphor-mutant (AA) or phosphor-mimic (SD) full-length human Tet3 constructs. Briefly, lentivirus was prepared by transfecting 293L cells with a mixture that contained 7 μg lentiviral plasmids (containing either empty vector, full-length Wt Tet3, AA Tet3 or SD Tet3) and Lenti-X packaging single shot reagent (VSV-G). 293L cells media harvested between 24 and 72 h after transfection was combined and concentrated using Lenti-X concentrator (Clontech) to produce lentiviral supernatant. Both *Tet1, 2* and *3* TKO and *Tet3* KO mouse ESCs were infected with lentiviral supernatant in the presence of polybrene (12 μg/ml). After 48 h of viral transduction, stable clones were selected using puromycin at a concentration of 0.1 μg/ml. For *Tet1, 2* and *3* TKO mouse ESCs, Wt25, AA10, Wt38 and AA35 lines with comparable level of Tet3 expression were isolated. For *Tet3* KO mouse ESCs, Wt14, AA13, Wt1, AA6, Wt2, AA3 and SD1 lines were isolated. Supplementary Table S7A contains detailed description of these different ESC lines.

### Dot blot assay

Zeta-probe membrane (Bio-rad) was hydrated with water and set up on a Bio-Dot apparatus (Bio-rad 1706545). Serial dilution of genomic DNA dissolved in 400 μl of 0.4 N NaOH was loaded into each well and rinsed twice with 0.4 N NaOH using vacuum suction. Membrane was rinsed briefly in 2× SSC buffer, UV-crosslinked for 10 min and subjected to standard immunoblot procedure using either 5mC, 5hmC or 5caC antibodies. The antibodies used are listed in Supplementary Table S9.

### RNA isolation and sequencing

Total RNA was isolated from ∼2 million of Wt25 and AA10 Tet3 mouse ESCs (*Tet1, 2* and *3* TKO) with RNAzol^®^RT (Sigma) according to manufacturer protocol. Total RNA samples from biological replicates were sent to BGI (Hong Kong) for high-throughput sequencing according to company's protocol. Briefly, the RNA quality was assessed with Agilent 2100 bioanalyzer. Following DNAse I treatment, mRNA enriched by magnetic beads coupled to Oligo-dT was fragmented and used as template for cDNA library construction. Short DNA fragments were then subjected to end-repair, A-tailing, adaptor ligation, size selection and sequenced on Illumina HiSeqTM4000.

### 5hmC-DNA IP

5hmC-DNA IP was carried out with according to published protocol (41) with minor modifications. Briefly, genomic DNA isolated by overnight lysis (100 mM Tris–HCl pH 8.0, 200 mM NaCl, 10 mM EDTA, 0.5% SDS, 50 μg proteinase K) and phenol/chloroform extraction was fragmented to 200–400 bp using Covaris M220 (PIP50, Duty 20%, cycles/burst 200, Time 200 s). Five micrograms of DNA fragments was subjected to end repair (NEB E6050), A-tailing (NEB M0212L) and adaptor ligation (NEB M0202M). The adaptor-ligated DNA was denatured by 10 min of boiling. Approximately 0.4 μg of DNA was set aside as input and 4 μg of DNA was diluted to 500 μl with 5× IP buffer (final concentration of 10 mM Na phosphate pH 7.0, 140 mM NaCl, 0.05% Triton). IP was performed by overnight incubation with 5 μg of anti-5hmC antibody (Active motif 39791) at 4°C. On the following day, 30 μl of pre-blocked (0.5% BSA in PBS) Protein G Dynabeads (Life Technologies 10003D) was added to the DNA-antibody complex. After 2 hr of incubation at 4°C, Dynabeads was captured on magnetic stand and washed 4 times with IP buffer. IP DNA was eluted from Dynabeads with 500 μl of digestion buffer (50 mM Tris pH 8.0, 10 mM EDTA, 0.5% SDS, 100 μg of proteinase K) for 4 h at 55°C. Input and IP DNA was purified by phenol/chloroform and ethanol precipitation. Barcoded libraries was then generated by KAPA real-time library amplification kit (KAPA Biosystems) and sequenced on Illumina HiseqTM4000 platform (Illumina 50 bp Pair-end) at BGI (Hong Kong).

### Quantification of 5hmC by *MspI* digestion

The differential level of 5hmC at specific loci was quantified using EpiMark® 5hmC and 5mC analysis kit protocol (E3317S, New England Biolabs) with minor modifications. This level was quantified based on the inhibition of *MspI* digestion at CCGG by glucosylated 5hmC. Briefly, 3 μg of genomic DNA purified from biological replicates of *Tet1, 2* and *3* TKO, WT25 and AA10 mouse ESCs was incubated with T4 Phage β-glucosyltransferase for 18 h at 37°C. After heat-inactivation, the glucosylated DNA was divided equally into: (i) digested by *MspI* (100 units) for 16 h at 37°C and (ii) an uncut control. The treated DNA samples were extracted by phenol–chloroform and ethanol precipitation. Two pairs of primers were designed for each locus of interest: (a) one pair that flanked the CCGG site and (b) the other pair that annealed to adjacent sequences with no CCGG site to serve as loading control. The 5hmC level was quantified by normalizing the CT values from (a) CCGG with (b) adjacent no CCGG reactions.

### RNA-sequencing analysis

Two biological replicates from Wt25 and phosphor-mutant AA10 Tet3 mESCs were subjected to RNA-seq. The quality of the raw reads was accessed using FastQC. Reads were aligned to Ensembl mouse genome (version GRCm38.87) using STAR (with the following arguments –outFilterMultimapNmax 1 –outSAMstrandField intronMotif –outSAMunmapped None). Read counts were generated using featureCounts from Rsubread Biconductor R package. Differential expression analysis was performed using DESeq2 with the differentially expressed genes (DEG) defined by FDR corrected *P*-value <0.05 and fold change ≥±1. The DEG were then overlapped with genes that contained DhMRs. The relative expression level of all genes was determined by Cufflinks and specified as fragments per kilobase per million (FPKM) (42). Expression heatmap of DEG was generated using ComplexHeatmap R package.

### Gene ontology enrichment analysis

Gene Ontology of genes which contained DhMRs and differentially expressed between wild-type and phosphor-mutant Tet3 mESCs was performed using the Database for Annotation, Visualization and Integrated Discovery (DAVID). GO terms with *P*-value <0.05 (Fisher Exact test with FDR correction) were considered significant and plotted using ggplot.

### 5hmC data processing and analysis

FastQC (https://www.bioinformatics.babraham.ac.uk/projects/fastqc/) was used to assess the quality of the sequencing reads. Raw sequencing reads from biological triplicates were mapped to the Ensembl mouse genome (version GRCm38.87) using STAR (with the arguments -alignEndsType EndToEnd –alignIntronMax 1 –outFilterMultimapNmax 1 –outFilterMatchNminOverLread 0.8). PCR duplicates were removed using Picard MarkDuplicates.

Quality control such as saturation analysis and CpG enrichment was carried out with functions from MEDIPS R/Bioconductor package (MEDIPS.saturation and MEDIPS.CpGenrich) to determine whether the number of reads is sufficient to generate a saturated and reproducible coverage profile of the reference genome as well as CpG enrichment across the DNA fragments (43).

Differential 5-hydroxymethylated regions (DhMRs) between Wt25 and AA10 Tet3 mouse ESCs were determined from the uniquely aligned reads using MEDIPS. MEDIPS divides the genome equally into bins with fixed window size (200 bp) and calculates the read counts across each bin. DhMRs were defined in bins with minimal number of reads counts and ≥1-fold change in the read counts between Wt25 and phosphor-mutant AA10 cells (*P* < 0.05). The DhMRs where the level of 5hmC is either higher in Wt25 (blue) or higher in AA10 (orange) mouse ESCs were annotated using Homer annotatePeaks.

To remove off-target binding of 5hmC-DIP (44), IgG-DIP of mouse ESCs datasets (SRX266835, SRX266836, SRX266847 and SRX266848) were downloaded (45). IgG-DIP enriched peaks with respect to its input were identified by MACS2 (*P* = 1e^−5^ and bw = 200). DhMRs with ≥1 bp overlap with IgG-DIP peaks were first filtered off to remove off-target binding sites. This was followed by the removal of DhMRs that contained simple tandem repeats (STR) and low complexity sequences.

### Correlation plot

The genome coverage tracks of 5hmC-DIP (WT25 and AA10) and IgG-DIP (ESCs) aligned reads files were generated using bamCoverage (deepTools). Genome coverage is calculated as the number of reads per 100 bp bin and normalized as Reads Per Kilobase per Million (RPKM). The average RPKM scores across the DhMRs were determined by multiBigwigSummary from deepTools. The correlation scatter plots were generated for different 5hmC-DIP (Wt25 and AA10 ESCs) and IgG–DIP (ESCs) samples using plotCorrelation (deepTools).

### Motif discovery analysis

Motif enrichment analysis of DNA fragments that contained 5hmC were carried out using MEME-ChIP (46). Matrix-clustering within the RSAT suite (http://rsat.eu/) (47) was used to build a complete motif tree which partitioned similar motifs into distinct clusters. Transcription factors associated with the individual motif were determined by Tomtom motif comparison tool using mouse Hocomoco (v11 FULL) database. The differential H3K27ac binding sites between Wt25 and AA10 cells were determined by DiffReps. Genes with differentially bound H3K27ac were then annotated using Homer annotatePeaks. The promoter regions (±1 kb of TSS) of these genes were subjected to motif analysis using MEME-ChIP.

### Immunofluorescence

Neurons cultured in 12-well plates were fixed with 4% paraformaldehyde in PBS for 20 min at room temperature. Cells were gently rinsed twice with 1× PBS and incubated with blocking solution (10% normal goat serum, 0.1% triton-X 100 in PBS) for 1 h at room temperature. This was followed by overnight staining with anti-MAP2 antibody (5 μg/ml in blocking buffer, Millipore) at 4°C. After three gentle washes with PBS, the neurons were incubated with anti-mouse 488 (1:500, Jackson ImmunoResearch). Following three washes with PBS, cells were mounted in VECTASHIELD with DAPI (Vector labs) and images were captured using Olympus (IX71, 20× objective) and confocal microscopes (FV3000, 10× objective). Images were analyzed using Imaris software (version 9.2.0, Bitplane). At least three fields were captured for each condition. The signal threshold was adjusted to obtain optimal coverage of the MAP2 positive soma and neurites of the neurons. The number of nuclei stained by DAPI was counted, which ranged from 1789 to 12240 per condition. The relative density was calculated as total MAP2 positive area over the number of nuclei stained by DAPI. The statistical significance was tabulated using the relative density from individual field for each condition. Detailed information of the quantification is summarized in Supplementary Table S7B.

## RESULTS

### Cdk5 phosphorylates Tet3 at a highly conserved serine residue

**Figure 1.**
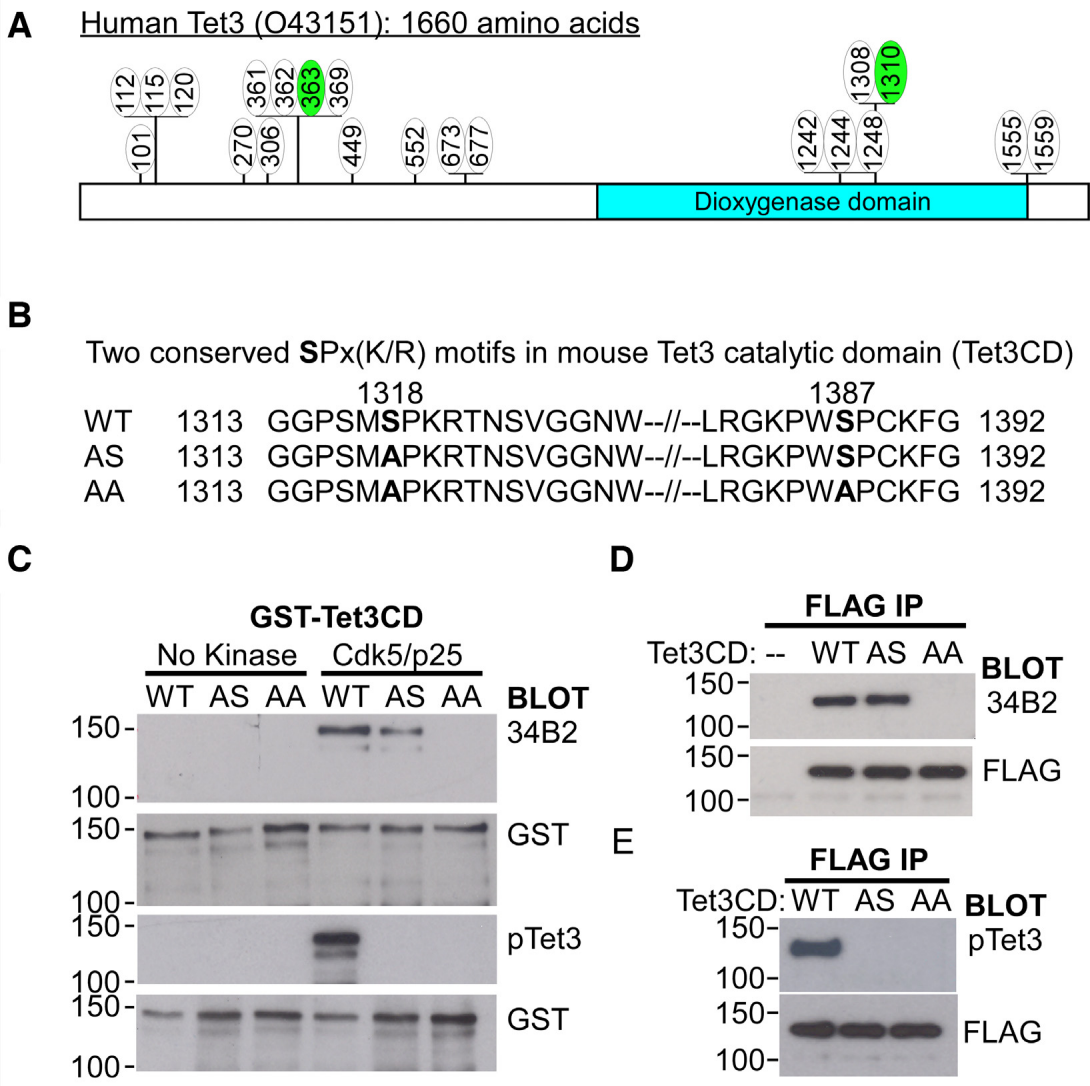
The catalytic domain of Tet3 is phosphorylated by Cdk5. (**A**) Schematic depiction of 21 phosphorylation sites on human Tet3 that were identified by mass spectrometry analysis. The green circles denote two conserved serine residues that were also phosphorylated in mouse Tet3 protein. (**B**) Amino acid sequences of the two conserved SPx(K/R) motifs found in the catalytic domain (CD) of mouse Tet3 protein, the single (AS) and double (AA) alanine mutants generated. (**C**) Phosphorylation of mouse Tet3CD by Cdk5 at S1318 and S1387 residues *in vitro*. GST-tagged wild-type (WT), single (AS) and double (AA) alanine mutant Tet3CD proteins were subjected to *in vitro* kinase assay in the presence or absence of Cdk5/p25. The products were probed with antibodies against GST, phosphor-serine with SPx(K/R) motif (34B2) and phosphorylated S1318 (pTet3). (**D, E**) Mouse Tet3 is phosphorylated at both S1318 and S1387 residues *in vivo*. Lysates from HEK293 cells transfected with either Flag-tagged WT, SA or AA Tet3CD constructs were immunoprecipitated (IP) and probed with FLAG, phosphor-serine 34B2 and pTet3 antibodies. Untransfected cells (–) were used as negative control.

To uncover novel post-translational modifications, full-length human Tet3 protein was transiently expressed and purified from HEK293 cells by immunoprecipitation (IP). Mass spectrometry analysis identified 21 phosphorylation sites, of which the conserved serine 363 and 1310 were previously reported to be phosphorylated in mouse Tet3 (Figure 1A and Supplementary Table S1.1) (33). Serine 1310 contains a SPx(K/R) motif that is highly conserved across different species and computationally predicted to be a substrate of cdk (Supplementary Figure S1A and Table S1.2). Interestingly, there are DNA polymorphisms that may disrupt this motif in human population (Supplementary Figure S1C). The overlapping roles of Tet3 and cdk5 in regulating various aspects of brain development (48) led us to postulate that this conserved serine might be a substrate of cdk5 in different species. To test this hypothesis and validate the report of mouse Tet3 phosphorylation (33), we performed *in vitro* kinase assay using bacterial purified GST-tagged mouse Tet3 catalytic domain (Tet3CD) (Figure 1B). Although the catalytic domain of both mouse and human Tet3 protein contains two highly conserved SPx(K/R) motifs (Figure 1B and Supplementary Figure S1B), mass spectrometry analyses could only detect phosphorylation at S1310 (human) and S1318 (mouse) from the first motif (Figure 1A). We generated wild-type (WT), single (AS) and double (AA) serine to alanine mutant mouse Tet3 proteins (Figure 1B). Phosphorylation was determined using commercially available 34B2 antibody which recognizes phosphor-serine (S*) within the S*Px(K/R) motif. In the presence of cdk5/p25, 34B2 antibody was able to detect the phosphorylated product in WT and AS, but not AA Tet3, indicating that both S1318 and S1387 were phosphorylated by cdk5 *in vitro* (Figure 1C). A phosphor-antibody that specifically recognizes the conserved phosphorylated serine (mouse S1318 and human S1310) of Tet3 was then generated (herein pTet3 antibody) (Supplementary Figure S1A). In accordance, this pTet3 antibody could only detect *in vitro* phosphorylated product in WT but not AS or AA mutant Tet3 protein (Figure 1C).

To test the specificity of pTet3 antibody *in vivo*, transiently expressed Flag-tagged WT, AS and AA mouse Tet3 proteins were pull-down from HEK293 cells. Contrary to result from mass spectrometry analysis (33), the 34B2 antibody readily detected phosphor-serine product in both WT and AS mutant Tet3 (Figure 1D). This indicates that mass spectrometry analysis might have failed to detect phosphorylation at S1387 of the second SPx(K/R) motif. Alternatively, S1387 may be phosphorylated to compensate for the loss of S1318 in the AS mutant (Figure 1D). Consistent with the *in vitro* data, the pTet3 antibody exhibited high specificity for phosphorylated S1318 *in vivo* (Figure 1E).

**Figure 2.**
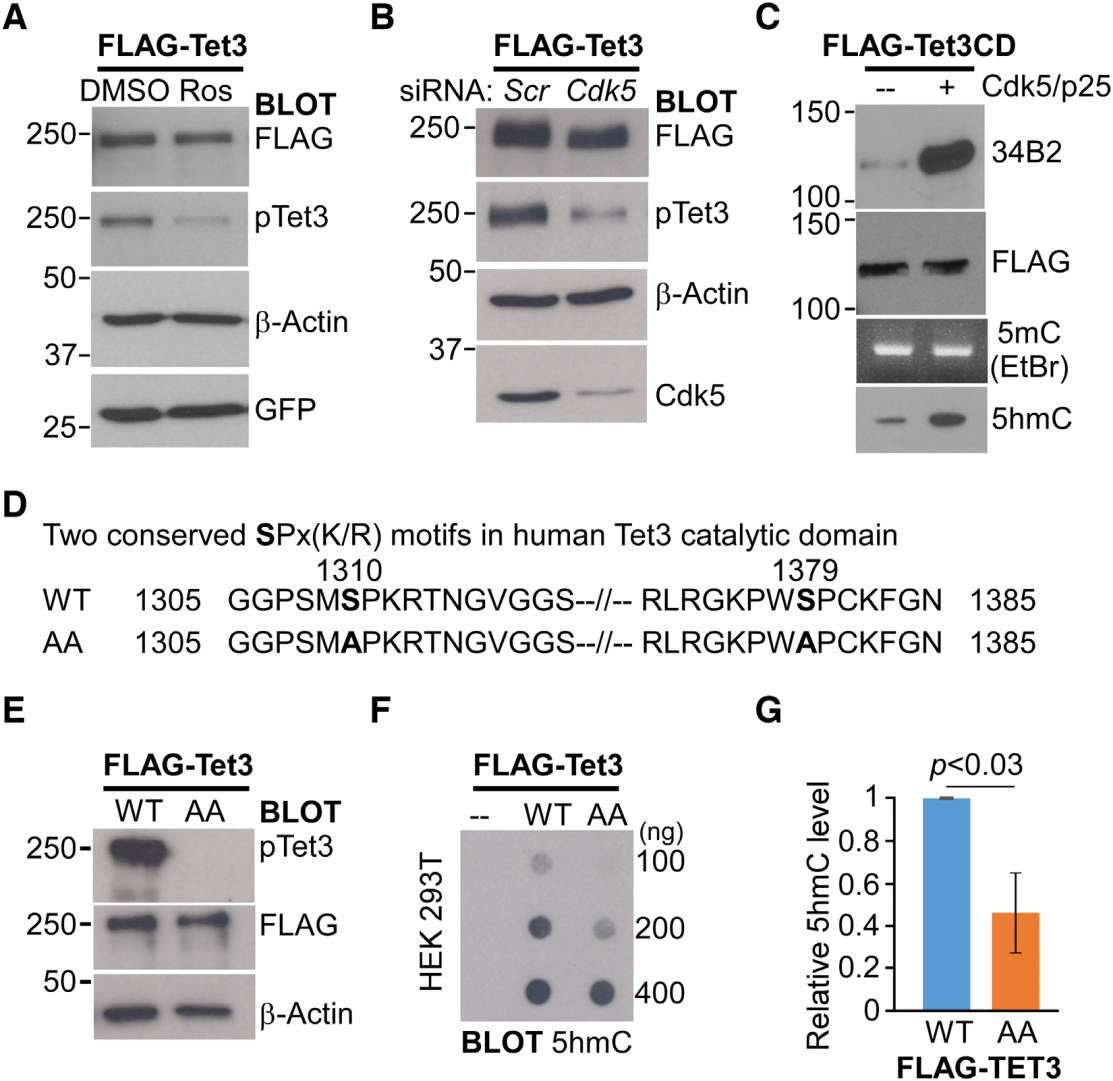
Cdk5-mediated phosphorylation of Tet3 increases its *in vitro* catalytic activity. (A. B) Cdk5 phosphorylates human Tet3 protein at S1310 *in vivo*. (**A**) HEK293 cells expressing Flag-tagged human Tet3 was treated with either DMSO or 20 μM of roscovitine (Ros) for 24 h. (**B**) HEK293 cells were co-transfected with Flag-tagged human Tet3 and either scrambled (scr) or Cdk5 siRNA for 48 hr. Cell lysates were western blotted with FLAG, pTet3, β-actin, GFP and Cdk5 antibodies. (**C**) Phosphorylated mouse Tet3CD exhibits increased catalytic activity *in vitro*. Flag-tagged mouse Tet3CD protein was subjected to *in vitro* kinase assay in the presence or absence of Cdk5/p25. One third of the proteins was western blotted with FLAG and 34B2 antibodies (Top). Another one third of the proteins were used for *in vitro* dioxygenase assay with equal amount of 5mC DNA. The DNA products were visualized by ethidium bromide (EtBr) staining on 2% agarose gel, Southern blotted and probed with 5hmC antibody (Bottom). (**D**) Amino acid sequences showing the two conserved SPx(K/R) motifs in the catalytic domain of human Tet3 protein and the double alanine (AA) mutants generated in this study. (E–G) Alanine substitution at S1310 and S1379 of human Tet3 protein leads to lower 5hmC level in HEK293 cells. (**E**) FACS sorted HEK293 cells that expressed either wild-type (WT) or phosphor-mutant (AA) full-length Flag-tagged human Tet3 were lysed and immunoblotted with pTet3, FLAG and β-actin antibodies. (**F**) Genomic DNA from untransfected HEK293 (–) and FACS sorted cells that expressed either WT or AA Flag-tagged human Tet3 were dot blotted and probed with 5hmC antibody. (**G**) Quantification of 5hmC level in transfected HEK293 cells where data are mean ± S.E.M. (*n* = 3).

We next tested if cdk5 is required for Tet3 phosphorylation by transiently expressing Flag-tagged full-length human Tet3 in HEK293 cells followed by 24 hr of treatment with either DMSO or roscovitine (Ros). Roscovitine is a broad-range purine analog inhibitor that blocks CDK1, 2, 5, 7 and 9 (with IC_50_ value of 0.1–0.7 μM), but not CDK4, 6 and 8 (49). There was significant reduction in the level of phosphorylated S1310 upon drug inhibition (Figure 2A). Cells were next co-transfected with Flag-tagged Tet3 and siRNA against cdk5. Compared to scrambled siRNA (*Scr*), siRNA against cdk5 led to significant reduction in the level of cdk5 protein and the concomitant decrease in the level of phosphorylated S1310 (Figure 2B). Taken together, our results indicate that cdk5 is both sufficient and necessary to phosphorylate Tet3 protein (Figures 1C and 2B).

As the conserved serine residues lie within the catalytic domain (CD), we asked if enzymatic activity of the Tet3 protein is affected by cdk5-mediated phosphorylation. Flag-tagged mouse Tet3CD purified from HEK293 cells was phosphorylated by cdk5 (Figure 2C, top). The unmodified or phosphorylated Tet3 protein was then incubated with equal amount of 5mC DNA for *in vitro* 5-hydroxylmethylation assay. While unmodified Tet3CD weakly oxidized 5mC into 5hmC, phosphorylation led to a higher rate of 5hmC conversion (Figure 2C, bottom), suggesting that phosphorylation may enhance Tet3 catalytic activity *in vitro*. To test if this is also observed in the cells, HEK293 cells were transiently transfected with different mouse Tet3CD constructs (Supplementary Figure S1D). Consistent with *in vitro* data, WT Tet3 exhibits higher 5hmC level than double phosphor-mutants AA Tet3 (Supplementary Figure S1E). Similar level of 5hmC induced by WT and AS (S1318A, S1387) mouse Tet3 suggests that phosphorylation of the second SPx(K/R) motif is equally important for Tet3 catalytic activity. Unexpectedly, phosphor-mimic (S1318D) Tet3 exhibits similar level of 5hmC as WT Tet3. In addition, WT Tet3 also led to higher 5caC (Supplementary Figure S1F) and lower 5mC (Supplementary Figure S1G) in cells when compared to AA Tet3. These results support the notion that non-phosphorylated Tet3 (i.e. AA) exhibits lower demethylation activity when transiently expressed in HEK293 cells.

To confirm if human Tet3 is regulated similarly by phosphorylation, we expressed either full-length Flag-tagged human wild-type (WT) or phosphor-mutant (AA) Tet3-ires-GFP constructs in HEK293 cells (Figure 2D). FACS sorted cells showed similar level of Tet3 proteins 48 h after transfection (Figure 2E). As a negative control, there was no detectable level of 5hmC in non-transfected HEK293 cells by dot-blot assay (Figure 2F). Consistent with the results from using mouse Tet3CD, the global level of 5hmC was significantly higher in cells that expressed WT human Tet3 cells than phosphor-mutant AA Tet3 (Figure 2F and G). Taken together, these results indicate that the regulation of the Tet3 catalytic activity by cdk5-mediated phosphorylation is highly conserved between mouse and human.

### Phosphorylated Tet3 leads to differential 5hmC patterns and transcriptional programs

**Figure 3.**
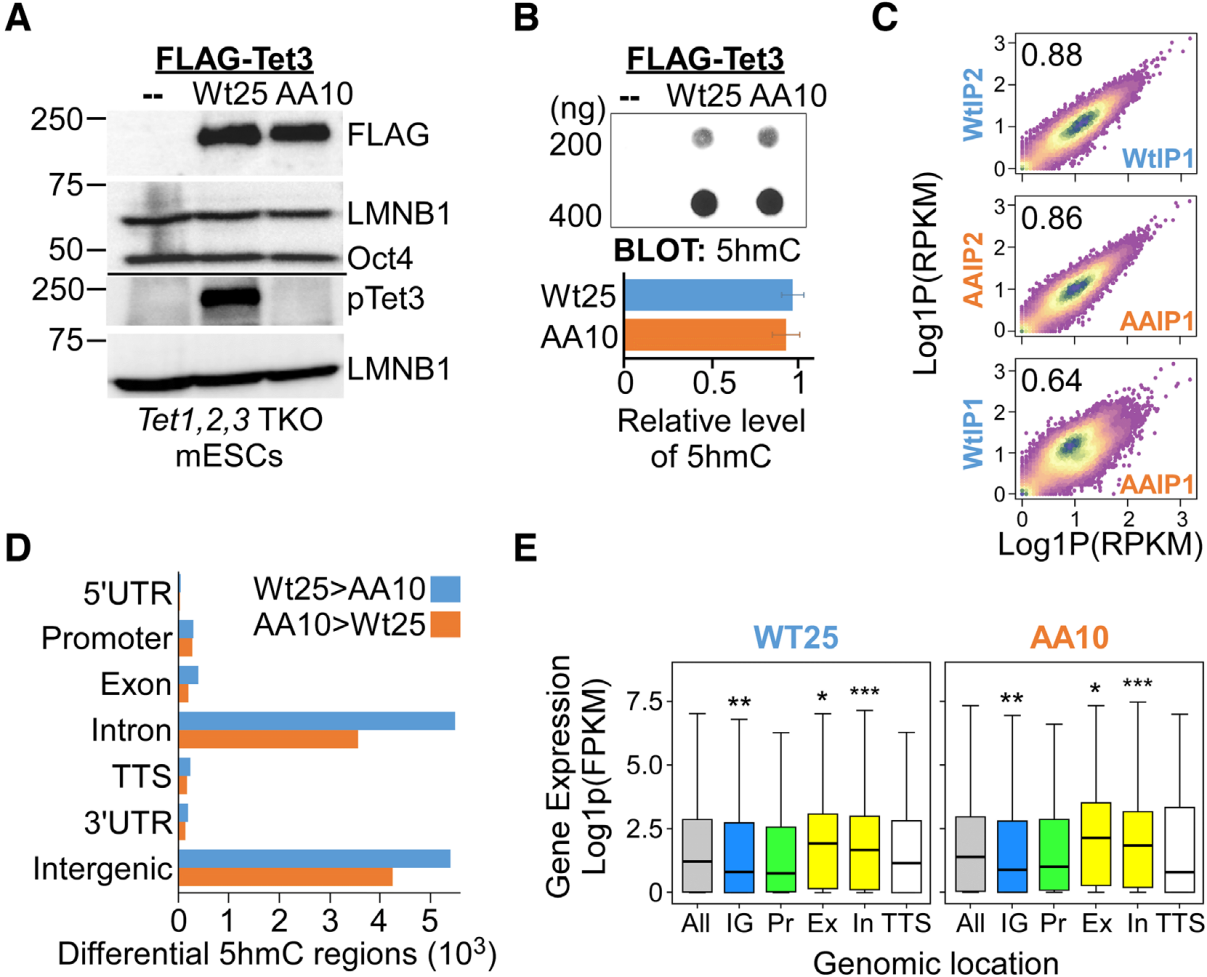
Correlation of gene expression with 5hmC enrichment at gene bodies in ESCs that expressed either wild-type or phosphor-mutant Tet3. (**A**) Stable *Tet1, 2, 3* triple knockout (TKO) mouse ESC lines that expressed comparable level of either Flag-tagged wild-type (Wt25) or double phosphor-mutants (AA10) human Tet3 were generated by lentiviral infection. Lysates from parental (–), Wt25 and AA10 were immunoblotted with FLAG, pTet3, Lamin B1 (LMNB1) and Oct4 antibodies. (**B**) Dot blot revealed no significant difference in the global 5hmC level between Wt25 and AA10 mouse ESC lines. Parental *Tet* TKO line (–) was used as negative control (Top). Quantification data are mean ± S.D. (*n* = 4) (bottom). (**C**) Density plot showing high reproducibility in 5hmC-DNA immunoprecipitation (DIP) signals across differential 5hmC regions (DhMRs) between two biological replicates from the same genotype, but lower Pearson correlation between Wt25 and AA10 cells. RPKM, reads per kilobase million mapped reads. (**D**) Genomic distribution of the annotated peaks of differential 5hmC regions between Wt25 and AA10 mouse ESCs. Blue bars indicate peaks where the 5hmC level is higher in Wt25 cells whereas orange bars indicate peaks where the level of 5hmC is higher in AA10 cells. UTR, untranslated region; TSS, transcription start site; TTS, transcription termination site. (**E**) Boxplots of mRNA expression of all genes (gray); genes with the presence of higher 5hmC differential level in the intergenic region (blue, IG); at the promoter (green, Pr); at the gene bodies (yellow, Ex: exons, In: introns) and at the TTS (white) in Wt25 and AA10 cells. Promoters were defined as ± 1kb from the TSS. Thick lines indicate mean and whiskers extend to ±1.5 of the interquartile range. Significance levels were calculated by Wilcoxon signed-rank test relative to ‘all genes’ category. WT: * *P* = 0.0003, ** *P =* 9.8e–5, *** *P* = 2.8e–8. AA: * *P* < 0.008, ** *P* = 7e–5, *** *P* = 1.9e–8. FPKM, fragments per kilobase million mapped reads.

To better understand the biological significance of cdk5-mediated phosphorylation and natural polymorphism at the SP(X)K site of human Tet3 (Supplementary Figure S1C), Flag-tagged full-length human wild-type and phosphor-mutant (AA: S1310A, S1379A) Tet3 were introduced into *Tet1*, *2*, *3* triple knockout (TKO) mouse ESCs (39) by lentiviral transduction. Independent lines with similar expression of Tet3 and pluripotency marker Oct4 were selected by puromycin (Figure 3A and Supplementary Figures S2A). 5hmC was undetectable in the TKO mouse ESCs, indicating the complete ablation of endogenous *Tet1*, *2* and *3* activity in these cells (Figure 3B, top). However, there was no difference in the global 5hmC level between *Tet1*, *2*, *3* TKO mouse ESCs expressing either wild-type (Wt) or AA Tet3 (herein Wt25 and AA10 mouse ESC lines) (Figure 3B, bottom). The discrepancy from earlier results could be due to the use of catalytic domain in *in vitro* assay (Figure 2C) or the transient expression of mouse and human *Tet3* gene in HEK293 cells (Supplementary Figure S1E and Figure 2G).

Instead of affecting the global level of 5hmC, it is plausible that cdk5-mediated phosphorylation of Tet3 may fine-tune the conversion of 5mC to 5hmC at specific gene loci. To test this hypothesis, the genomic-wide 5hmC profiles from three biological replicates of Wt25 and AA10 mouse ESCs were generated by 5-hydroxymethylated cytosine DNA immunoprecipitation (5hmC-DIP) coupled with high-throughput sequencing. We obtained approximately 82–86 million of total uniquely mapped paired-end reads of 5hmC-enriched sequences for each genotype (Supplementary Table S2). Differentially 5-hydroxymethylated regions (DhMRs) between Wt25 and AA10 mouse ESCs were next identified using MEDIPS algorithm (43). With a cut-off of *P* < 0.05 and fold change ≥1, MEDIPS identified approximately 13078 and 9115 regions where the level of 5hmC is higher in Wt25 cells and AA10 cells respectively. A recent study highlighted the importance to normalize 5hmC-DIP data using control IgG-DIP to remove off-target binding of short unmodified DNA elements like the simple tandem repeats (STR) and low complexity sequences (44). Therefore, we removed DhMRs that have at least one base-pair overlap with IgG-DIP binding sites (45) as well as STR and low complexity sequences (Supplementary Figure S2B, Table S3). The 5hmC profiles are highly reproducible among the biological triplicates from the same genotype at these DhMRs (Pearson correlation ≥ 0.8, Figure 3C and Supplementary Figure S2C), but exhibit lower correlation with IgG-DIP (Supplementary Figure S2D). Majority of the filtered DhMRs are localized to the intergenic and intronic regions (Figure 3D, Supplementary Figure S2E and Table S3). The relative frequency of CpGs across the DhMRs is approximately two fold higher than the reference genome sequences (Supplementary Table S4). Several of the identified DhMRs were next validated by either 5hmC-DIP RT-PCR or *MspI* restriction digest (50). Undetectable or low RT-PCR signals from IgG-DIP and 5hmC-DIP of *Tet1, 2* and *3* TKO ESCs genomic DNA indicated that the DhMRs identified in our study are highly specific to 5hmC modification (Supplementary Figure S3A and S3B). *MspI* digestion is inhibited by glucosylated 5hmC moiety at CCGG site (50). Importantly, RT-PCR analysis of *MspI* treated genomic DNA from Wt25 and AA10 cells demonstrated the presence of differential 5hmC level at several of the tested DhMRs (Supplementary Figure S3C–E). Using MEME-ChIP package to examine the sequences of DhMRs (46), we identified 40 transcription factors binding motifs in the regions where 5hmC was higher in Wt25 cells and 33 motifs in regions with higher 5hmC in AA10 cells (Supplementary Table S4). These motifs were grouped hierarchically into 23 distinct clusters with RSAT matrix-clustering tool (Supplementary Figure S4, Table S4) (47). Interestingly, the motifs with highly similar DNA sequences in some clusters appeared to be recognized by distinct transcription factors. For instance, in cluster 13 and 16, regions with higher 5hmC level in Wt25 cells contain motifs that are recognized by ZKSC1 and RARB, whereas regions with higher level of 5hmC in AA10 cells contain binding motifs for FOXP2 and RARG. This suggests that wild-type and phosphor-mutant Tet3 may elicit DhMRs at distinct transcription factors binding sites.

**Figure 4.**
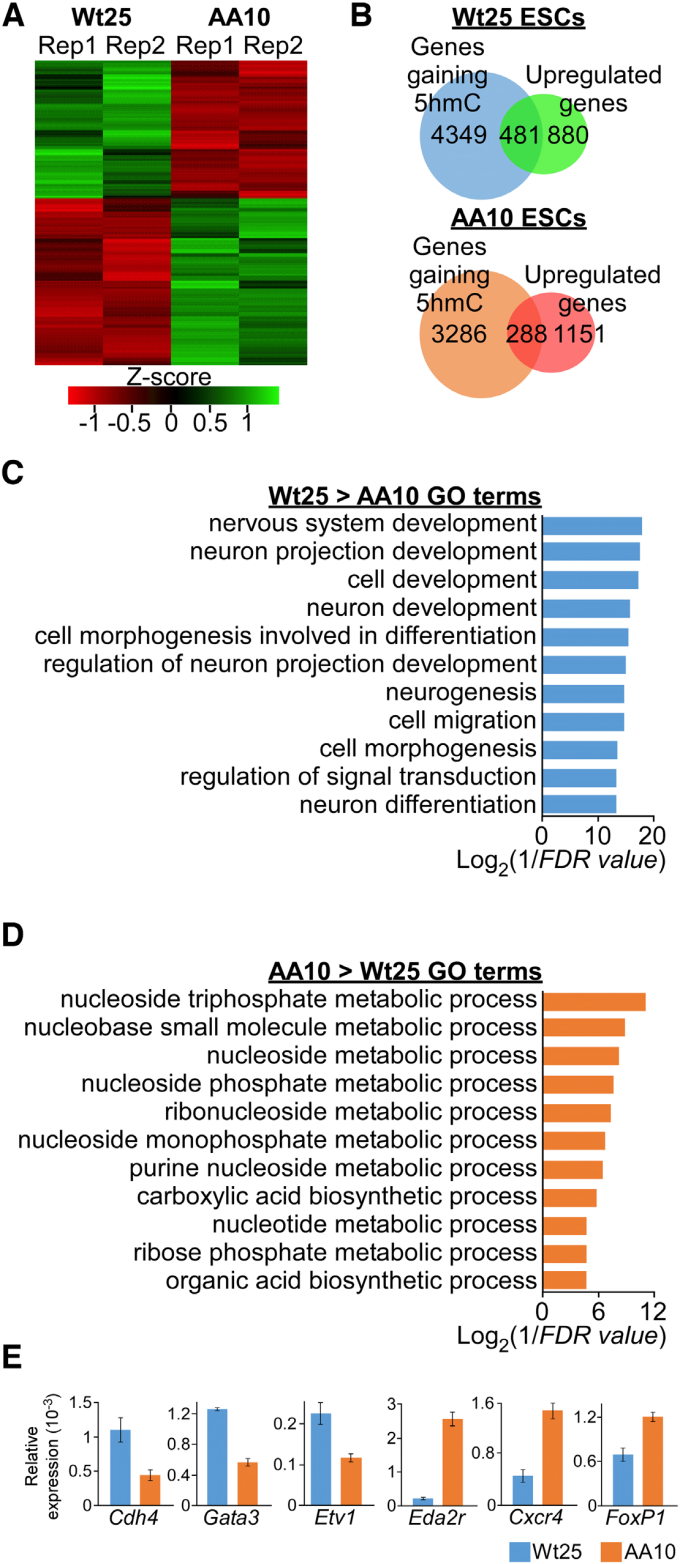
Phosphorylation at S1310 and S1379 leads to higher 5hmC and expression of neuronal genes. (**A**) Heatmap of differentially expressed genes that were determined by DEseq2 (adjusted *P*-value < 0.05) between two biological replicates (Rep1 and Rep2) of Wt25 and AA10 mouse ESCs. (**B**) Venn diagrams showing the overlaps in the number of genes that have higher level of 5hmC and upregulation of mRNA expression in Wt25 (top) and AA10 (bottom) mouse ESCs. (**C**) Enriched GO terms for genes with higher level of 5hmC and mRNA expression in Wt25 compared to AA10 mouse ESCs. (**D**) Enriched GO terms for genes with higher level of 5hmC and mRNA expression in AA10 compared to Wt25 mouse ESCs. (**E**) Validation of differentially expressed genes in Wt25 and AA10 mouse ESCs by quantitative PCR (qPCR) using *Gapdh* expression as reference. Representative data are presented as mean ± S.D. (triplicate qPCR reactions). Similar expression pattern was observed in biological replicate as well as in independent mouse ESCs lines (Supplementary Figure S5).

To better understand how the differential levels of 5hmC may influence gene expression and the chromatin state, we performed RNA-sequencing and native chromatin immunoprecipitation (ChIP) with H3K27ac antibody in Wt25 and AA10 ESCs. Principal component analysis of the aligned RNA-seq reads revealed high reproducibility between two biological replicates of Wt25 and AA10 cells (Supplementary Figure S5A). Similar to other studies (7–9,11), enrichment of higher 5hmC level in the gene bodies (exon and introns) are associated with higher gene expression in the respective cells (Figure 3E). Pairwise comparison of RNA-seq data with DESeq2 (adjusted *P*-value < 0.05, fold change ≥ 1) revealed 1361 and 1439 genes that were up-regulated in Wt25 and AA10 cells respectively (Figure 4A and Supplementary Table S5). Many of these genes were further validated by quantitative RT-PCR in independent ESCs lines (Supplementary Figure S5B and S5C, Wt25/AA10 and Wt38/AA35, Supplementary Table S7A). Several hundreds of the differential expressed genes also overlapped with the increased DhMRs in each cell line, suggesting that the phosphorylation of Tet3 may regulate the level of 5hmC and gene expression patterns at specific loci (Figure 4B and Supplementary Table S5). Interestingly, gene ontology (GO) analysis showed that genes with increased level of 5hmC and mRNA expression in Wt25 cells are involved with various neuronal developmental processes (Figure 4C, Supplementary Table S6). On the other hand, genes with higher 5hmC and expression in AA10 cells are mainly associated with different metabolic processes (Figure 4D, E and Supplementary Table S6). Several of the differentially expressed neuronal genes (Supplementary Table S6) were validated experimentally by quantitative RT-PCR in independent ESCs lines (Figure 4E and Supplementary Figure S5C). Taken together, our data suggests that phosphorylation of Tet3 might be necessary for optimal activation of neuron-specific programs during differentiation.

Histone modification H3K27ac, which defines both active enhancers and promoters, is enriched at the TSS/promoter, intronic and intergenic regions in both Wt25 and AA10 ESCs (Supplementary Figure S5D). However, increased mRNA expression was only observed for genes with H3K27ac peaks at their promoters or gene bodies (Supplementary Figure S5E). 5hmC has been demonstrated to be associated with the poised enhancers in mouse ESCs (7,10–12), but may define a subset of active enhancers that trigger gene expression during cardiomyocytes differentiation (16). Similar expression pattern was observed at genes that harbour H3K27ac mark and DhMRs, although there is a substantial drop in the expression of genes with lower 5hmC level in AA10 cells (Supplementary Figure S5F). We next asked if genes with differential H3K27ac level between Wt25 and AA10 cells may contain distinct transcription factor binding motifs at their promoters. Aside from several common transcriptional activators (e.g. SP2, ATF1, ERG and WT1), motif discovery analysis identified several cell-type specific motifs within the promoters of genes with differential H3K27ac level. There was an enrichment for RBP-J (Notch signalling) and MSX3 (involved in neurogenesis) binding motifs at regions where H3K27ac is higher in Wt25 cells (Supplementary Figure S5G). On the other hand, at sites where H3K27ac is higher in AA10 cells, there was enrichment for REST (inhibitor of neurogenesis) and NRF1 (metabolic pathways) binding motifs (Supplementary Figure S5G). As Tet3 has been shown to bind Notch target genes during neuronal differentiation (23), this result suggests that priming of Notch-responsive elements with H3K27ac mark might be regulated by phosphorylated Tet3.

### Phosphorylation of Tet3 ensures efficient neuronal differentiation

**Figure 5.**
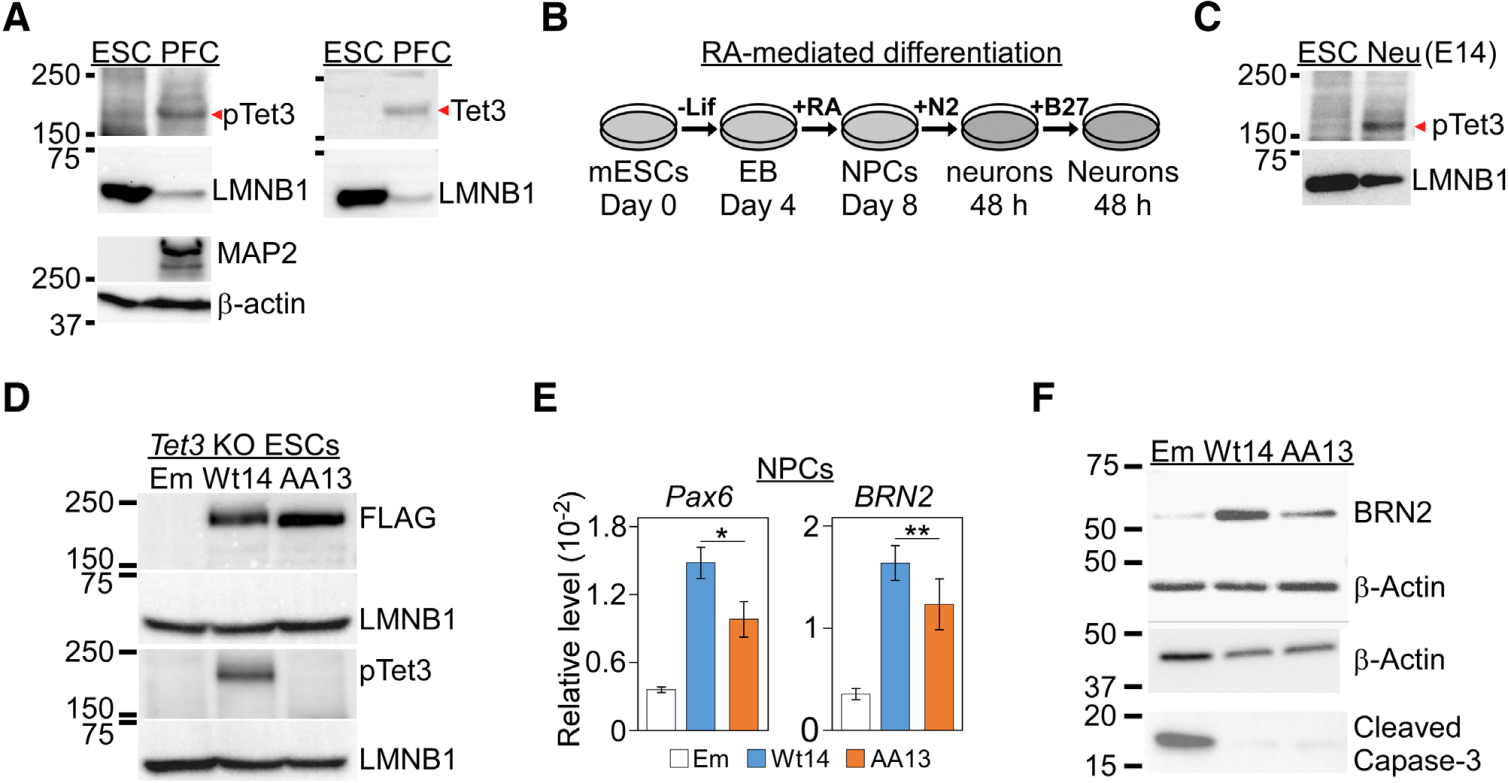
Phosphor-mutant Tet3 ESCs show impaired neuronal differentiation. (**A**) Detection of S1318 phosphorylation of endogenous Tet3 in the prefrontal cortex (PFC) tissues from *C57BL/6* mice but not in mouse E14 ESC. (**B**) Retinoic acid (RA) was used to induce *in vitro* neuronal differentiation of mouse ESCs. Lif, Leukemia inhibitory factor; EB, embryoid bodies; NPC, neuronal progenitor cells; +N2 and +B27 indicate the addition of the respective culture medium for the indicated duration (48 h). (**C**) Detection of S1318 phosphorylation of endogenous Tet3 in neurons that were differentiated from E14 ESC. Neurons were cultured in N2 medium for 48 h. (**D**) Generation of stable *Tet3* knockout (KO) mouse ESC lines that expressed either empty lentiviral vector (Em), Flag-tagged wild-type (Wt14) or double phosphor-mutants (AA13) human full-length *Tet3* gene. Lysates from were probed with FLAG, pTet3, Tet3, MAP2, β-actin and LMNB1 antibodies. (**E**) Expression of *Pax6 and BRN2* genes is significantly lower in *Tet3* KO NPCs that were rescued with phosphor-mutant AA Tet3. Wt14 and AA13 lines were subjected to qPCR using *GAPDH* as reference. Data are shown as mean ± S.E.M. (*Pax6*,*n* = 6, * *P* = 0.04; *BRN2*, *n* = 5, ** *P* = 0.014). (**F**) Representative western blot of lysates from Em, Wt14 and AA13 NPCs using BRN2, cleaved caspase-3 and β-actin antibodies.

To address the physiological relevance of our findings, we asked if endogenous Tet3 is phosphorylated at the conserved serine residue in the brain tissues as well as during neuronal differentiation of mouse E14 ESCs. Immunoblot with pTet3 and Tet3 antibodies showed the presence of phosphorylated Tet3 in the prefrontal cortex (PFC) tissues harvested from *C57BL/6* mice but not in E14 ESCs (Figure 5A). The E14 ESCs were then differentiated into neurons by retinoic acid (RA) treatment (Figure 5B) (40). The detection of pTet3 band in the neurons suggests that phosphorylation of endogenous Tet3 may be involved in regulating the differentiation of E14 ESCs (Figure 5C). To test this hypothesis, we expressed either wild-type or phosphor-mutant human Tet3 cDNA in *Tet3* knockout (KO) mouse ESCs by lentiviral infection. Three independent pairs of *Tet3* KO mouse ESCs lines with comparable expression level of either wild-type (Wt) or phosphor-mutant (AA) Tet3 were selected by puromycin treatment (herein Wt14/AA13, Wt1/AA6 and Wt2/AA3 lines) (Figure 5D, Supplementary Figure S6A and Table S7). The regulatory role of Tet3 phosphorylation was then evaluated by measuring the expression level of key neuronal genes such as *Pax6* and *BRN2*, as well as terminal neuronal marker MAP2 in the neurons differentiated from these ESCs lines.

Loss of Tet3 has been shown to impair *in vitro* neuronal differentiation (23) whereas its ectopic expression in the presence of Forskolin can drive neurogenesis of fibroblasts (25). Therefore, we asked if constitutive expression of wild-type Tet3 can induce ESCs to form neurons. *BRN2* encodes a key neuronal transcription factor that acts downstream of RA-mediated induction (51). In the absence of RA, wild-type *Tet3* expressing ESC (Wt14 line) were unable to activate *BRN2* or *Pax6* gene expression in the NPC stage (Supplementary Figure S6B) nor induce the formation of MAP2 positive neurons (Supplementary Figure S6B and S6C). This result indicates that constitutive expression of Tet3 in ESCs alone is insufficient to drive neuronal differentiation.

On the other hand, we observed modest yet statistically significant difference in the expression level of *Pax6* (*P* = 0.04, *n* = 6) and *BRN2* (*P* = 0.014, *n* = 5) genes between wild-type (Wt14) and phosphor-mutant (AA13) Tet3 NPCs (Figure 5E). This in turn led to lower level of BRN2 protein in phosphor-mutant AA NPCs (Figure 5F, top). Similar gene expression pattern was also observed in two independent pairs of wild-type and phosphor-mutant NPCs (Wt1/AA6 and Wt2/AA3; Supplementary Figure S6D). Consistent with earlier *in vitro* demethylase result (Supplementary Figure S1E), phosphor-mimic S1310D Tet3 expressing neurons (SD1) elicited similar level of *BRN2* expression as compared to wild-type Tet3 cells (Wt2) (Supplementary Figure S6D). Moreover, in line with the reported role of Tet3 in suppressing apoptosis (24), we observed elevated level of cleaved capase-3 in control (Em: Empty lentiviral vector) but not in wild-type or phosphor-mutant AA Tet3 expressing NPCs (Figure 5F, bottom). These results indicate that Tet3 phosphorylation is not necessary for activating neuronal genes expression or inhibiting apoptosis. Instead, it may provide a permissive epigenetic landscape to facilitate higher transcription of specific neuronal gene like *BRN2* at the NPCs stage.

**Figure 6.**
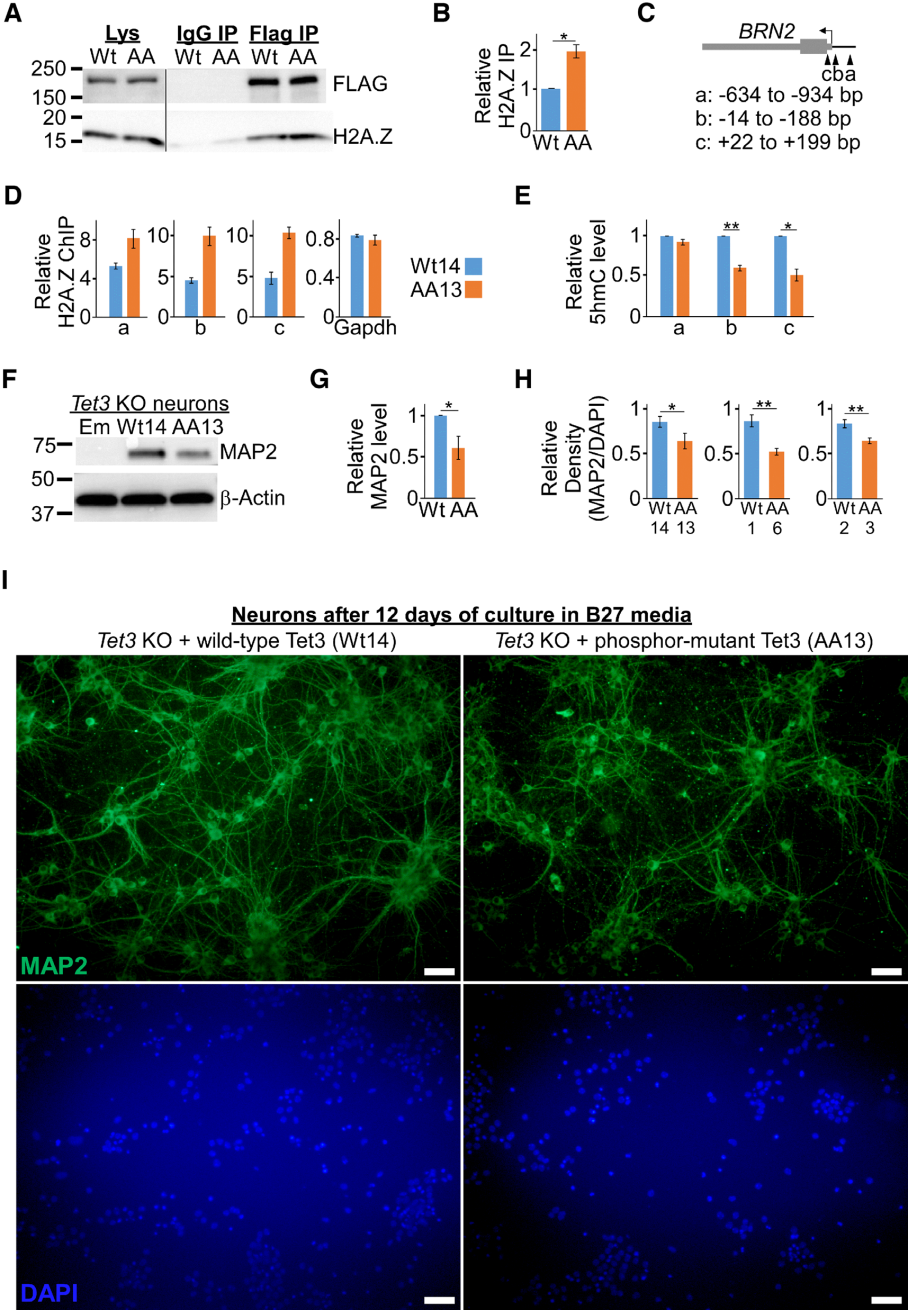
Impaired differentiation of phosphor-mutant NPCs is correlated with unique epigenetic signature and lower *BRN2* expression. (**A**) Phosphor-mutant (AA) Tet3 has higher binding affinity to histone H2A.Z in mouse ESCs. Lysates from *Tet3* KO mouse ESCs that expressed either wild-type (Wt) or phosphor-mutant (AA) Tet3 were IP with either IgG or FLAG antibodies. The IP factions were then probed for FLAG and H2A.Z. (**B**) Quantification of IP blots with data presented as mean ± S.E.M. (four replicates of Wt14/AA13; one replicate of Wt1/AA6 lines; * *P* = 0.003, paired one-tailed *t*-test). (**C**) Schematic of *BRN2* gene promoter that was reported to contain differentially DNA methylated sites and H2A.Z nucleosomes. (**D**) H2A.Z occupancy in AA13 is specifically higher at the *BRN2* promoter compared to Wt14 ESCs. Representative ChIP data are presented as mean ± S.D. (triplicate qPCR reactions, *P* < 0.05). Similar pattern was also observed in another biological replicate (Supplementary Figure S6F). (**E**) Reduced 5hmC level at *BRN2* gene promoter in phosphor-mutant (AA13) NPCs. The 5hmC level in Wt14/AA13 NPCs was determined with 5hmC-DIP followed by qPCR and presented as mean ± S.E.M. (*n* = 4, * *P* = 0.004, ** *P* = 0.0007, paired one-tailed *t*-test). (**F, G**) Phosphor-mutant Tet3 (AA13) line has lower level of MAP2 protein in terminally differentiated neurons. (**F**) Representative western blot of neurons that were cultured in B27 medium for 48 h and probed with MAP2 and β-actin antibodies. (**G**) Quantification of four independent experiments with data represented as mean ± S.E.M. (*n* = 4, * *P* < 0.04, paired one-tailed *t*-test). (**H**) Quantification of the density of MAP2-positive neurons after long-term culture in B27 medium. Data are presented as mean ± S.E.M. (Wt14/AA13, 12 days of culture, Figure 6I, * *P* < 0.04; Wt1/AA6, 13 days of culture, Supplementary Figure S8; Wt2/AA3, 8 days of culture, Supplementary Figure S9; ** *P* < 0.003, unpaired one-tailed *t*-test. See also Supplementary Table S7B for detailed analysis). (**I**) Wt14 *Tet3* KO mouse ESCs line differentiated efficiently to form higher density of MAP2-positive neurons when compared to AA13 line. Same number of NPCs were seeded and neurons were stained with MAP2 antibody after 12 days of culture in B27 medium.

To gain insights into the regulatory mechanisms of wild-type and phosphor-mutant AA Tet3, we examined the proteins that co-IP with human Tet3 protein from our initial mass spectrometry analysis (Supplementary Table S1.3). In addition from well-characterized interacting partners like O-GlcNAc transferase, histone variant H2A.Z was found to co-IP with Tet3 protein. Given that H2A.Z regulates transcriptional activation (52) and is important for neurogenesis (53), we asked if H2A.Z may have differential binding affinity to wild-type and phosphor-mutant AA Tet3 proteins. In Wt14 and AA13 *Tet3* KO mouse ESCs, FLAG but not immunoglobulin (IgG) antibody was able to pull-down a protein complex that contains Flag-tagged Tet3 and H2A.Z (Figure 6A). Interestingly, there is a considerable increase in the amount of H2A.Z protein (∼2 fold, *P* = 0.014, *n* = 5) that was pull-down by phosphor-mutant compared to wild-type Tet3 (Figure 6B). Consistent with this observation, we found that the level of H2A.Z occupancy at the *BRN2* promoter (Figure 6C and Supplementary Figure S6E) is significantly higher in AA13 when compared to Wt14 Tet3 KO mouse ESCs (Figure 6D and Supplementary Figure S6F). This appears to be locus-specific as there was no difference in H2A.Z occupancy at the promoter of *GAPDH* gene. The higher occupancy of H2A.Z would suggest a lower rate of nucleosomes eviction at *BRN2* promoter in AA13 mouse ESCs.

We next asked if wild-type and phosphor-mutant Tet3 may also lead to differential DNA methylation regions in the NPCs. There was no statistically significant difference in the global level of 5hmC between wild-type and phosphor-mutant AA Tet3 NPCs (Supplementary Figure S6G, *n* = 4). To address the possibility of locus-specific changes, 5hmC-DIP followed by qPCR was conducted at previously identified 5hmC-enriched regions (11,25) in Wt14 and AA13 Tet3 NPCs. For instance, *BRN2* promoter has been shown to undergo Tet3-mediated demethylation during neuronal differentiation (Supplementary Figure S6E) (25). Interestingly, we observed the higher level of 5hmC at the promoter of *BRN2* in Wt 14 cells (Figure 6E, *n* = 4). Tet3 has also been demonstrated to bind to *Hes1* and *Hey2* genes that are downstream targets of Notch signalling pathways during neuronal differentiation (23). We also observed higher level of 5hmC in the intronic region of *Hey2* (Supplementary Figure S6H, bottom, *P* = 0.005, n = 3) gene in Wt14 NPCs that correlates with its mRNA expression. There was however no difference in the level of 5hmC at *Hes1* gene between wild-type and phosphor-mutant AA NPCs (Supplementary Figure S6H, top, *n* = 3). Taken together, our data suggests that phosphorylation of Tet3 may lead to higher level of 5hmC and lower H2A.Z occupancy at *BRN2* promoter, which could in turn induce higher rate of gene transcription.

The lower expression of *Pax6* and *BRN2* genes suggests that phosphor-mutant NPCs may differentiate less efficiently than their wild-type counterparts. To test this, we seeded the same number of NPCs from three independent wild-type and phosphor-mutant *Tet3* KO lines (Wt14/AA13, Wt1/AA6, Wt2/AA3). This was followed by examining the expression level of terminal neuronal marker MAP2 and the morphology of the differentiated neurons at different time-points in culture (Figure 5B). The level of MAP2 protein was highest in Wt14 neurons, significantly lower in AA13 neurons (*n* = 4) and barely detectable in control (Em) neurons after 48 hours culture in B27 medium (Figure 6F and G). Similar pattern of MAP2 protein expression was also observed in Wt1/AA6 and Wt2/AA3 neurons (Supplementary Figure S7A). However, in line with the *BRN2* expression in NPCs, there was no observable difference in the level of MAP2 protein between wild-type (Wt2) and phosphor-mimic S1310D (SD1) line.

Consistent with the level of MAP2 protein, wild-type lines (Wt1 and Wt2) have more MAP2 positive neurons compared to phosphor-mutant lines (AA6 and AA3) after 48 hours culture in B27 medium (Supplementary Figure S7B, C and D). After prolonged culture in B27 medium (8–13 days), wild-type Tet3 lines produced higher density of MAP2 positive neurons than phosphor-mutant Tet3 lines (Figure 6H). For instance, Wt14 line formed higher density of MAP2-positive neurons that have more extensive neurite outgrowth when compared to AA13 line after 12 days of culture (Figure 6I). Similarly, Wt1/Wt2 lines have higher density of neurons compared to AA6/AA3 lines after 8 to 13 days of culture (Supplementary Figures S8 and S9). Taken together, these results support the notion that phosphorylation of Tet3 at S1310 and S1379 residues is required for robust activation of neuron-specific transcription factor like BRN2, which in turn, promotes optimal terminal neuronal differentiation.

## DISCUSSION

Tet3 regulates neuronal differentiation and synaptic plasticity (23,24,26,27,54), but the mechanisms by which Tet3 is regulated to elicit distinct transcriptional outputs remain unclear. Here, we show that cdk5-mediated phosphorylation of Tet3 at S1310 and S1379 residues leads to strong activation of neuron-specific genes like *BRN2*. Conversely, phosphor-mutant Tet3 drives the expression of genes that are linked to different metabolic processes in mouse ESCs. Compared to the phosphor-mutant, wild-type Tet3 increases the level of 5hmC and H2A.Z eviction at the promoter of *BRN2* gene. Such epigenetic changes are in turn correlated with higher mRNA expression and robust neuronal differentiation of ESCs that expressed wild-type Tet3.

While phosphorylated Tet3 exhibits elevated catalytic activity *in vitro* and in transiently transfected HEK293 cells, there is no significant difference in the global 5hmC level of *Tet1, 2, 3* TKO mouse ESCs that stably expressed either wild-type (Wt25) or phosphor-mutant (AA10) Tet3 protein. Instead, our data indicate that both phosphorylated and phosphor-mutant Tet3 may induce differential 5hmC level at distinct sets of genes. While the underlying mechanism has not been fully elucidated, several inferences can be drawn from existing data.

First, the presence of unique DNA motifs within the differential 5hmC regions of Wt25 and AA10 cells postulates that wild-type and phosphor-mutant Tet3 may be recruited to the chromatin by different transcription factors. Consistent with this notion, it has been demonstrated that the activity of Tet enzymes may be modulated differently by their interacting partners like PGC7 (34), REST (54) and AR9 (55) at specific genomic locations. Interestingly, in regions where the level of 5hmC is higher in Wt25 cells, there is also enrichment of motifs for factors like MSX3 and RARB (Supplementary Figure S4B). As these factors are not highly expressed in ESCs, this observation suggests that phosphorylated Tet3 may selectively remove 5mC from these transcription factor binding sites to increase their accessibility during differentiation (56). Second, the activity of Tet enzymes is known to be regulated by its association to O-linked *N*-acetylglucosamine transferase (OGT) and the subsequent O*-*GlcNAcylation at the serine/threonine residues where phosphorylation may occur (32,33). The presence of phosphorylation in wild-type but not mutant Tet3 may lead to alteration in the level of glycosylation and interaction with OGT. Third, several recent studies indicate that phosphorylation may confer context-dependent functions to Tet proteins. For instance, ATR (Ataxia telangiectasia and Rad3-related protein)-mediated phosphorylation of Tet3 promotes changes in 5hmC patterns that is necessary for timely DNA damage responses (57) whereas *in vivo* tumour-suppressor activity of Tet2 is highly stabilized by AMPK (AMP-activated kinase)-mediated phosphorylation (58). In our study, phosphorylated and phosphor-mutant Tet3 exhibit differential binding affinity towards H2A.Z, which in turn affect H2A.Z occupancy at *BRN2* promoter region. The differential degree of H2A.Z eviction from regulatory elements could potentially affect the rate of transcription of *BRN2* gene (52).

In summary, we identify S1310 and S1379 phosphorylation of Tet3 protein as a critical regulatory mechanism that elicits optimal expression of neuron-specific genes during neuronal differentiation of mouse ESCs. However, it remains unclear if this modification regulates neural plasticity, differentiation of other neuronal subtypes and susceptibility to neurodegenerative disorder. This is of particular interest given the existence of natural polymorphism that could disrupt the phosphorylation motif in human Tet3 gene. Generation of a knock-in mouse model with serine substitution at this phosphorylation site will provide valuable tool toward investigating some of these questions.

## DATA AVAILABILITY

The GEO accession number for the sequencing data is GSE130384.

## Supplementary Material

gkz1144_Supplemental_Files
