# Supplementary material for: Phosphorylation of Tet3 by cdk5 is critical for robust activation of BRN2 during neuronal differentiation
Source: Nucleic Acids Res. 2019 Dec 6;48(3):1225–38. doi: 10.1093/nar/gkz1144 (PMC7026633; doi:10.1093/nar/gkz1144)
Supplement: gkz1144_Supplemental_Files [file gkz1144_supplemental_files.zip › Rao VK et al Supplemental data.pdf]

Supplementary Data

Inventory of Supplementary Tables

Table	*Name*	*Sheet or Part*
S1	Mass spectrometry of human Tet3	1) Different post-translational modifications on human Tet3<br>2) Ions data for phosphorylated S1310<br>3) Human Tet3 interacting protein partners in HEK293 cells
S2	Summary of NGS datasets	A) 5hmC-DNA-IP and H3K27ac ChIP<br>B) RNA-sequencing
S3	Differential 5hmC regions (DhMRs) identified in Wt25 & AA10 by 5hmC-DNA-IP	1) Overview of DhMRs (number & genomic features)<br>2) Wt25>AA10 Differential 5hmC regions<br>3) AA10>Wt25 Differential 5hmC regions<br>4) Annotated genes with (or nearest to) DhMRs<br>5) Filtered Wt25>AA10 DhMRs with simple repeats.<br>6) Filtered AA10>Wt25 DhMRs with simple repeats.
S4	Motif analysis of DhMRs	1) MEME analysis of Wt25 > AA10 DhMRs<br>2) MEME analysis of AA10 > Wt25 DhMRs<br>3) RSAT clustering of the motifs<br>4) CG content of DhMR versus genomic DNA<br>5) IgG-DIP MACS peaks
S5	DEG in Wt25 & AA10 mouse ESCs	1) Wt25 > AA10 ESCs mRNA transcripts<br>2) AA10 > Wt25 ESCs mRNA transcripts<br>3) Differential expression genes (DEG)<br>4) Overlap between DEG and genes with DhMRs
S6	GO term analysis of genes with increased 5hmC & expression in Wt25 & AA10 ESCs	1) GO term analysis of Wt25 > AA10 (DEG + DhMRs)<br>2) GO term analysis of AA10 > Wt25 (DEG + DhMRs)<br>3) Genes name from GO terms analysis (WT25>AA10)
S7	Summary of mouse ESCs lines generated<br><br>Quantification of neuronal differentiation	A) Summary of mouse ESC lines generated<br><br>B) Quantification of neuronal differentiation experiment
S8	Primer sequences	A) Molecular Cloning<br>B) qPCR validation of mRNA expression<br>C) 5hmC-DNA-IP and H2A.Z ChIP-qPCR<br>D) 5hmC-DIP RT-PCR and *MspI* validation
S9	Primers validation & Antibodies List	1) Validation of qPCR primers used for mRNA expression<br>2) Validation of 5hmC-DIP RT-PCR & *MspI* primers<br>3) List of antibodies used

Figures S1 to S9

Supplemental Material & Methods

Purification of recombinant proteins

GST-Tet3CD and Flag-TetCD proteins were purified from BL21(DE3)pLysS *E. coli* cells (Promega) and HEK293T cells using GST·Bind resin (Novagen) and anti-Flag M2 affinity gel (Sigma) respectively. Flag-TetCD proteins were eluted with Flag peptides (Sigma) and concentrated using Pierce concentrator 30K MXCO (Thermo Scientific). Flag-Tet3CD was used for *in vitro* phosphorylation assay whereas Flag-Tet1CD protein was used for Tab-seq.

Western blot analysis

For western analysis, proteins were transferred to PVSF membrane (Millipore) in Tris-glycine transfer buffer and 20% methanol for 2 hr at 100 V. The membranes were blocked 1 hr in TBST (20mM Tris, pH7.4, 150 mM NaCl, 0.05% Tween 20) with either 5% skimmed milk powder or 5% BSA (for phosphor-antibody), followed by overnight incubation with the specific primary antibody. The list of primary antibodies used are: rabbit-anti-H2A.Z (1:4000, Abcam ab188314), rabbit-anti-pTet3 (1:1000, Genscript), mouse-anti-Cdk5 (1:1000, Millipore), rabbit-anti-5hmC (1:1000, Active Motif), rabbit-anti-Oct4 (1:1000, Proteintech), rabbit-anti-lamin B1 (1:10000, Abcam); mouse-anti-MAP2 (1:500, A4) and mouse-anti-BRN2 (1:500, B2) (both Santa Cruz); rabbit-anti-Phospho-MAPK/CDK Substrates (PXS*P and S*PXR/K) (1:1000, 34B2) and rabbit-anti-cleaved caspase-3 (1:1000) (both Cell Signalling Technology); mouse-anti-Flag (1:2000), mouse-anti-actin (1:5000) and mouse-anti-GST (1:5000) (All from Sigma). Membranes were washed three times with TBST and probed with secondary antibodies-conjugated to HRP (1:5,000, Novex, Invitrogen) for 1 hr. After three more washes, the presence of different proteins was detected using SuperSignal West Pico/Dura Chemiluminescent substrate (Thermo Scientific). Quantification of Western blot data was carried out with ImageJ software. All the antibodies used are listed in Table S9.

Liquid chromatography with tandem mass spectroscopy (Normal LC/MS)

Protein resolved in the polyacrylamide gel was stained with EZblue^{TM} dye (Sigma) and the excised Flag-Tet3 protein fragment was submitted to Proteomic and mass spectrometry core facility, School of Biological Sciences at National Technological University for LC/MS procedure according to their protocol. Following tryspin treatment, the peptides were separated and analyzed using a Dionex Ultimate 3000 RSLCnano system coupled to a Q Exactive

instrument (Thermo Fisher Scientific). Separation was performed on a Dionex EASY-Spray 75 μm × 10 cm column packed with PepMap C18 3 μm, 100 Å (Thermo Fisher Scientific) using solvent A (0.1% formic acid) and solvent B (0.1% formic acid in 100% ACN) at flow rate of 300 nL/min with a 60 min gradient. Peptides were then analyzed on a Q Exactive apparatus with an EASY nanospray source (Thermo Fisher Scientific) at an electrospray potential of 1.5 kV. A full MS scan (350–1,600 m/z range) was acquired at a resolution of 70,000 and a maximum ion accumulation time of 100 msec. Dynamic exclusion was set as 30 sec. The resolution of the higher energy collisional dissociation (HCD) spectra was set to 35000. The automatic gain control (AGC) settings of the full MS scan and the MS2 scan were 5E6 and 2E5, respectively. The 10 most intense ions above the 2,000 count threshold were selected for fragmentation in HCD, with a maximum ion accumulation time of 120 msec. An isolation width of 2 m/z was used for MS2. Single and unassigned charged ions were excluded from MS/MS. For HCD, the normalized collision energy was set to 30%. The underfill ratio was defined as 0.3%. Raw data files were processed and searched using Proteome Discoverer 1.4 (Thermo Fisher Scientific). The Mascot algorithm was used for data searching to identify proteins using the following parameters; missed cleavage of two; dynamic modifications were oxidation (+15.995 Da) (M), phosphorylation (+79.966 Da) (S, T, Y) or Methyl 1 OH (+30.010 Da) (K, R). The static modifications was Carbamidomethyl (+57 Da) (C). The false discovery rate for protein identification was <1%.

Native chromatin immunoprecipitation with H3K27ac and H2A.Z antibody

The protocol was adapted from (1) with minor modifications. 2 million each of Wt25 and AA10 *Tet1, 2, 3* TKO mouse ESCs were centrifuged at 1000g for 5 min, washed once in PBS, frozen in liquid nitrogen and stored at -80°C until further use. Frozen cell pellet ($2x10^6$) was resuspended thoroughly in 400 μl of NBA buffer (85 mM NaCl, 5.5% sucrose, 10 mM Tris-HCl, pH 7.5, 0.2 mM EDTA, 0.2 mM PMSF, 1 mM DTT, 5mM sodium butyrate and 1× protease inhibitors, Roche) followed by equal volume of 0.1% NP-40 in NBA buffer. After 5 min of incubation on ice, nuclei were pelleted at 4,000*g* for 3 min at 4°C. The nuclear pellet was washed with 1 ml of NBR buffer (85 mM NaCl, 5.5% sucrose, 10 mM Tris-HCl, pH 7.5, 3 mM $MgCl_2$, 1.5 mM $CaCl_2$, 0.2 mM PMSF, 1 mM DTT, 5mM sodium butyrate and 1× protease inhibitors) and pelleted at 4,000*g* for 3 min at 4°C. Nuclei pellet was resuspended in 750 μl of NBR buffer supplemented with 20 μg of RNase A and incubated at 20°C for 5 min. Chromatin was fragmented using micrococcal nuclease at 37°C for 15 min (MNase Worthington, titrated to give 75% mononucleosomes: 25% dinucleosomes). Digestion was

stopped with the addition of an equal volume of STOP buffer (215 mM NaCl, 10 mM Tris-HCl, pH 8, 20 mM EDTA, 5.5% sucrose, 2% Triton X-100, 0.2 mM PMSF, 1 mM DTT, 5mM sodium butyrate and 1× protease inhibitors). The digested nuclei was sonicated for 10 seconds with ultrasonic processor (Vibra-Cell™ VC 130S at output watts of 6) and incubated on ice for 3-4 hours to release soluble chromatin. After centrifugation at 12,000*g* for 10 min at 4°C, the supernatant (soluble chromatin) was transferred to new eppendorf tube. Ten percent of soluble chromatin was retained as input, and the remainder was incubated with ~3 to 5 µg of antibody (H3K27ac, ab4729 or H2A.Z, Ab188314) overnight at 4°C on a rotating wheel. 25 µl of Protein-A Dynabeads (Life Technologies, 10002D) was washed once and blocked overnight in PBS containing 5 mg/ml BSA and 0.1 mM PMSF. On the next day, the blocking buffer was removed from Dynabeads. The chromatin/antibody mixture was added to Dynabeads and incubated for 4 hours with agitation at 4°C. The immune complexes bound to Dynabeads were washed five times with wash buffer (150 mM NaCl, 10 mM Tris-HCl, pH 8, 2 mM EDTA, 1% NP-40 and 1% sodium deoxycholate) on nutating mixer and once in room-temperature TE buffer for 10 min/wash. Chromatin was released from the Dynabeads by incubation with 0.1 M $NaHCO_3$ in 1% SDS for 30 min at 37°C followed by the addition of proteinase K (100 µg/ml) and Tris, pH 6.8 (100 mM), and overnight incubation at 55°C. 5 M NaCl was added to a final concentration of 0.2 M. DNA was extracted with phenol/chloroform and chloroform, followed by ethanol precipitation.

H3K27ac ChIP-seq data processing and analysis

The quality of the raw sequencing reads was assessed using FastQC and mapped to the Ensembl mouse genome (version GRCm38.87) using STAR (with parameters: --alignEndsType EndToEnd --alignIntronMax 1 --outFilterMultimapNmax 1 --outFilterMatchNminOverLread 0.8). Multiple mapped reads were marked and filtered out using the Picard MarkDuplicates. H3K27ac enriched peaks were identified using Homer findpeaks. Pooled peaks across replicates were annotated with Homer annotatePeaks and used for downstream analysis. Boxplots for expression of genes there were enriched in H3K27ac and/or DhMRs were generated using ggpubr in R.

Primers design

Primers were either sourced from the primer bank or designed using Primer3 suite and validated according to MIQE guidelines (2). PCR specificity was confirmed by running the amplicon on DNA agarose gel. PCR efficiency was determined by the slope and y-intercept in

the standard curve analysis. The primers sequences and their validation results are summarized in Tables S8 and S9 respectively.

Experimental validation by quantitative RT-PCR

For validation of differential gene expression, high capacity cDNA reverse transcription kit (Applied Biosystems) was used to generate cDNA libraries from 1 µg of total RNA that was pretreated with DNase I (Thermo scientific). For quantification of 5hmC-DIP, the concentration of input and IP DNA was measured by Qubit fluorometer (Invitrogen) and diluted accordingly.

Quantitative real-time PCR was carried out in triplicates (6 µl/reaction) with 0.2 µM of primers and 2x SYBR green master mix (Thermo Scientifc) using 7900 HT real-time PCR machine (Applied Biosystems). Relative gene expression was calculated using $2^{-\Delta CT}$ method where threshold cycle (CT) values obtained from target genes were normalized to *GAPDH* gene. For 5hmC-DIP, the level of IP was calculated relative to its input DNA.

Supplementary Figure legend

Figure S1. Human Tet3 protein is phosphorylated at the highly conserved S1310 and S1379 residues

(A-B) Tet3 catalytic domain (CD) contains two highly conserved SPxR motifs that undergo phosphorylation at the serine residues (highlighted in red). **(A)** The phosphorylated serine identified by mass spectrometry analysis is highlighted by green circle. The green line denotes the 15 amino acids used as epitope to generate phosphor-serine pTet3 antibody.

(C) Naturally occurring missense DNA polymorphisms (annotated by Exome Aggregation Consortium) can disrupt the conserved SPxR motif in human population.

(D) The wild-type (WT: S1318, S1387) and different mouse Tet3CD mutants used for dot blot analysis. Single phosphor-mutant (AS: S1318A), double phosphor-mutant (AA: S1318A, S1387A) and phosphor-mimic (SD: S1318D) Tet3CD.

(E) Dot blot showing the global 5hmC level of unsorted HEK293 cells that were transiently transfected with either WT or different mouse Tet3CD mutants. Untransfected HEK293 cells (--) was used as a control.

(F-G) Dot blot analysis and quantification of HEK293 cells that expressed either WT or different mouse Tet3CD mutants. **(F)** Mouse Tet1CD induced significantly higher level of 5caC compared to Tet3CD (left). WT exhibited modestly higher level of 5caC than AA Tet3CD (right). **(G)** WT exhibited modestly lower level of 5mC compared to AA Tet3CD. * $P < 0.05$ (n = 2, paired 1-tailed *t*-test).

Figure S2. Identification of differential 5hmC regions (DhMRs) between Wt25 and AA10 ESCs

(A) Immunoblot of two independent pairs of *Tet1, 2, 3* triple knockout (TKO) mouse ESCs that expressed either Flag-tagged wild-type or double phosphor-mutants human Tet3 (Wt25/AA10 and Wt38/AA35 lines). Mouse ESCs lysates were probed with FLAG, pTet3, β-Actin, Cdk5 and Oct4 antibodies.

(B) DhMRs identified by MEDIPS on three replicates of 5hmC-DIP-seq were filtered to remove off-target IgG-DIP binding sites, simple tandem repeats (STR) and low complexity sequences.

(C) High reproducibility in 5hmC-DIP signals across the DhMRs between two biological replicates of the same genotype, but lower Pearson correlation between Wt25 and AA10 samples. RPKM, reads per kilobase million mapped reads.

(D) Correlation plot between 5hmC-DIP-seq of genomic DNA from Wt25 and AA10 ESCs versus IgG-DIP of genomic DNA from ESCs.

(E) Integrated Genomic View (IGV) profile of 5hmC-DIP signals from three biological replicates of Wt25 and AA10 mouse ESCs at *Cdh4* gene. The DhMRs determined by MEDIPS were depicted as green/grey (Wt25 > AA10) bars and magnified to illustrate the differential 5hmC signals.

Figure S3. Validation of DhMRs by 5hmC-DIP RT-PCR and *MspI* assay

(A-B) 5hmC-DIP signals from three replicates of Wt25 and AA10 samples at **(A)** *Cdh4* and **(B)** *FoxP1* gene (left panel). The DhMRs determined by MEDIPS (3) were depicted as grey bars. Locus-specific RT-PCR showed low or undetectable level of signal from IgG-DIP and 5hmC-DIP of genomic DNA isolated from *Tet1, 2* and *3* TKO ESCs, but significant differences between Wt25 and AA10 (right panel).

(C-E) 5hmC-DIP signals from three replicates of Wt25 and AA10 samples at **(C)** *Cdh4*, **(D)** *MAPT* and **(E)** *Elovl6* gene (left panel). Genomic sequences of the DhMRs which contain the CCGG site. *MspI* digestion is inhibited by glycosylated (G) 5hmC (middle panel). RT-PCR showed low level of signal from *MspI* digested DNA isolated from *Tet1, 2* and *3* TKO ESCs but statistically significant differences between Wt25 and AA10 samples (right panel). Data are presented as mean ± S.D. and represent two independent biological experiments. *P*-value was calculated by two-tailed *t*-test.

Figure S4. DhMRs contain unique transcription factor binding motifs.

(A) There are 40 motifs (blue) that underlie the regions where 5hmC is higher in Wt25 cells and 33 motifs (orange) associated with regions where 5hmC is higher in AA10 cells. These motifs were grouped into 23 clusters based on their sequence similarity.

(B) Examples of TF that may recognize the motif in each cluster. ID refers to the assigned motif ranking in MEME analysis.

Figure S5. Validation of differentially expressed genes and characterization of differential H3K27ac patterns

(A) Principal component analysis (PCA) of aligned RNA-sequencing reads from two biological replicates of Wt25 and AA10 mouse ESCs.

(B-C) Quantitative RT-PCR validation of selected differentially expressed genes in **(B)** Wt25/AA10 and **(C)** Wt38/AA35 mouse ESCs lines. Relative level was calculated using

Gapdh expression as reference. Representative data are presented as mean ± S.D. (triplicate qPCR reactions). Similar pattern was also observed in biological replicate (data not shown).

(D) Genomic distribution of H3K27ac enriched peaks that were identified in Wt25 and AA10 mouse ESCs by HOMER software.

(E) Enrichment of H3K27ac at the gene body and promoter is correlated with higher gene expression. Boxplots of RNA expression of all genes (grey); genes with H3K27ac peaks in the gene body (yellow, GB); H3K27ac at the nearest intergenic region (blue, Int) and at the promoter (green, P). Promoters were defined as ± 1kb from the TSS. Thick lines indicate mean and whiskers extend to ±1.5 of the interquartile range. Significance levels were calculated by Mann-Whitney test relative to "all genes" category. ** P-value < 2.2e-16.

(F) Boxplots of RNA expression for genes with H3K27ac marks ± WT>AA or AA>WT DhMRs in Wt25 (blue) and AA10 (orange) mouse ESCs. There is no significance difference in the gene expression patterns between different groups except WT>AA DhMRs in AA10 ESCs. P-value is calculated by Wilcoxon signed-rank test.

(G) Association of different transcription factor binding motifs within the promoter regions (± 1kb TSS) of genes that have differential H3K27ac level between Wt25 and AA10 mouse ESCs.

Figure S6. Tet3 phosphorylation leads to robust expression of neuronal genes

A) Immunoblots of independent *Tet3* knockout (KO) mouse ESCs lines that expressed either empty lentiviral vector (Em), Flag-tagged wild-type (Wt), double phosphor-mutants (AA) or phosphor mimic mutant (SD) human Tet3. The different ESCs lines were paired as follow: Em/Wt14/AA13, Wt1/AA6 and Wt2/AA3/SD1. Cellular lysates were probed with Flag, pTet3, Oct4, LMNB1 and β-actin antibodies.

(B-C) Constitutive expression of Tet3 alone in ESCs is insufficient to induce neuronal differentiation. *Tet3* KO mouse ESCs that expressed wild-type Tet3 (Wt14 line) were differentiated in the presence or absence of RA. **(B)** Detection of *BRN2* and *Pax6* mRNA in NPCs (top) and MAP2 protein in differentiated neurons (bottom) only upon RA induction. **(C)** Immunofluorescence imaging revealed the formation of MAP2-positive neurons only in the presence of RA. Black and white scale bar depicts 50 μm.

(D) Expression of *Pax6 and BRN2* genes is significantly higher in *Tet3* KO NPCs that expressed wild-type (Wt) compared to phosphor-mutant (AA) Tet3. Data are shown as mean ± S.E.M. (*Pax6*: Wt1/AA6, n = 2, ** $p = 0.02$; *BRN2*: Wt1/AA6, n = 4, *** $p = 0.005$; *BRN2*: Wt2/AA3, n = 3, * $p = 0.04$).

(E) Distribution of H2A.Z over the promoter of *GAPDH* and *BRN2* genes in mouse ESCs based on previous study (GSM2683432) (4). Sites "*a*", "*b*" and "*c*" have been shown to undergo Tet3-mediated demethylation during neurogenesis (5).

(F) H2A.Z occupancy in AA13 is higher at the *BRN2* promoter compared to Wt14 ESCs. ChIP data are presented as mean ± S.D. from triplicate qPCR reactions (second biological replicate for Fig.6D).

(G) Dot blot assay revealed no significant difference in level of 5hmC in the NPCs that were differentiated from *Tet3* KO mouse ESCs expressing either wild-type (Wt) or phosphor-mutant (AA) human Tet3.

(H) Higher *Hey2* expression in Wt14 NPCs is correlated to the intronic (int-3) level of 5hmC. Relative expression of Notch target genes *Hes1* and *Hey2* was determined by qPCR. The 5hmC level in Wt14/AA13 NPCs was determined using 5-hmC-DIP followed by qPCR. Data are presented as mean ± S.E.M. (* $p < 0.04$, n = 5; ** $p = 0.005$, n = 3; paired 1-tailed *t*-test).

Figure S7. *Tet3* KO ESCs that expressed phosphor-mutant (AA) Tet3 form less MAP2+ neurons after 2 days culture

(A) Phosphor-mutant (AA) Tet3 lines expressed lower level of MAP2 protein in terminally differentiated neurons. Neurons differentiated from various mouse ESCs lines (Wt1, AA6; Wt2, AA3 and SD1) were cultured for 48 h in B27 medium and immunoblotted with MAP2 antibody.

(B) *Tet3* KO ESCs that expressed phosphor-mutant (AA) Tet3 formed lower density of MAP2+ neurons after 48h culture in B27 medium. Data are presented as mean ± S.E.M. (Wt1/AA6, Fig.S7C, * $p = 0.002$; Wt2/AA3, Fig.S7D, ** $p = 0.0001$, unpaired 1-tailed *t*-test).

(C-D) Representative immunofluorescence staining of MAP2+ neurons after 48 h of culture in B27 medium. Neurons differentiated from **(C)** Wt1 and AA6 ESCs lines, as well as **(D)** Wt2 and AA3 ESCs lines. White bar indicates 50 μm scale.

Figure S8. Reduced efficiency of neuronal differentiation of phosphor-mutant Tet3 (AA6) ESCs

(A-B) *Tet3* KO mouse ESCs expressing wild-type Tet3 (Wt1) differentiated efficiently to form extensive neurites outgrowth when compared to phosphor-mutant Tet3 (AA6) ESCs. Representative immunofluorescence staining of differentiated MAP2+ neurons after 13 days culture in B27 medium. **(A)** High and **(B)** low magnification images with white bar indicating 50 μm scale. These images were used for the quantification of neuron density (Fig.6H).

Figure S9. Reduced efficiency of neuronal differentiation of phosphor-mutant Tet3 (AA3) ESCs

Tet3 KO mouse ESCs expressing wild-type Tet3 (Wt2) differentiated efficiently to form higher density of MAP2+ neurons when compared to phosphor-mutant Tet3 (AA3) ESCs. Same number of NPCs were seeded and neurons were stained with MAP2 antibody after 8 days of culture in B27 medium. White bar indicates 50 μm scale. These images were used for the quantification of neuron density (Fig.6H).

Supplementary Figures

Figure S1

A

UniProtKB	Species	Protein Sequences
O43151	Human	1292 --QNGGPSHLWGQYSGGPSMSPKRTNGVGGSWGVFSSGE--
H2QI55	Chimpanzee	1314 --QNGGPSHLWGQYSGGPSMSPKRTNGVGGSWGVFSSGE--
K7EUU4	Sumatran orangutan	1427 --QNGGPSHLWGQYSGGPSMSPKRTNGVGGSWSVFSSGE--
G7NAD1	Rhesus macaque	1327 --QNGGPSHLWGQYSGGPSMSPKRTNSVGGSWGVFSSGE--
F7AKG6	Horse	1293 --QNGGPNHLWGQYSGGPSMSPKRTNSVGGSWGVFSSGE--
F1PBJ1	Dog	1429 --QNGGPNHLWGQYSGGPSMSPKRTNSVGGSWGMFHPGE--
M3WR93	Cat	1334 --QNGGPNHLWGQYSGGPSMSPKRTNSVGGSWGVFHPGE--
S7PMY6	Brandt's bat	1291 --QNGGPNHLWGQYSGGPSMSPKRTNSVGGSWGVFPPGE--
D3ZQT7	Rat	1434 --QNGGPGHLWGQYSGGPSMSPKRTNSVGGNWGVFPSGE--
Q8BG87	Mouse	1300 --QNGGPSHLWGQYSGGPSMSPKRTNSVGGNWGVFPPGE--
		***** ******************.***.*.:* **

15 amino acids used to generate pTet3 antibody

B

UniProtKB	Species	Protein Sequences
O43151	Human	1357 --LFPGEGQQAASHSGGRLRGKPWSPCKFGNSTSALAGP--
H2QI55	Chimpanzee	1379 --LFPGEGQQVASHSGGRLRGKPWSPCKFGNSTSALAGP--
K7EUU4	Sumatran orangutan	1492 --LFPGEGQQAASHSGGRLRGKPWSPCKFGNSTSALAGP--
G7NAD1	Rhesus macaque	1392 --LFPGEGQQAASHSGGRLRGKPWSPCKFGNSTSTLTGP--
F7AKG6	Horse	1358 --LFPGEGQQPAPQPGGRPRGKPWSPCKFGNSTAALAGP--
F1PBJ1	Dog	1494 --LFSGEGQQPAPQPGGRLRSKPWSPCKFGNTTSALAGP--
M3WR93	Cat	1399 --LFPGEGQQPAPQPGGRLRSKPWSPCKFGNNTSALAGP--
S7PMY6	Brandt's bat	1356 --LFPGEGQQLAPQPGGRLRGKPWSPCKFGNNTSALAGP--
D3ZQT7	Rat	1499 --LFTGEGQQSAPHPGGRLRGKPWSPCKFGNGTSALSGP--
Q8BG87	Mouse	1365 --LFTGEGQQSAPHAGARLRGKPWSPCKFGNGTSALTGP--
		** ***** * : *.* *.********** *::*:**

C

Human Tet3

```
Chromosome 2:74328248 - TCC CCC AAG AGG - 7432859
Amino Acids:      1310 - S   P   K   R   - 1314
```

Polymorphism	Amino Acid Substitution
1) 74328249 C/T	Serine 1310 to Phenylalanine
2) 74328251 C/G	Proline 1311 to Alanine
3) 74328251 C/T	Proline 1311 to Serine
4) 74328254 A/C	Lysine 1312 to Glutamine

D

Mouse Tet3 constructs

	1318 / 1387
WT	-SPKR-//-SPCK-
AA	-APKR-//-APCK-
AS	-APKR-//-SPCK-
SD	-DPKR-//-SPCK-

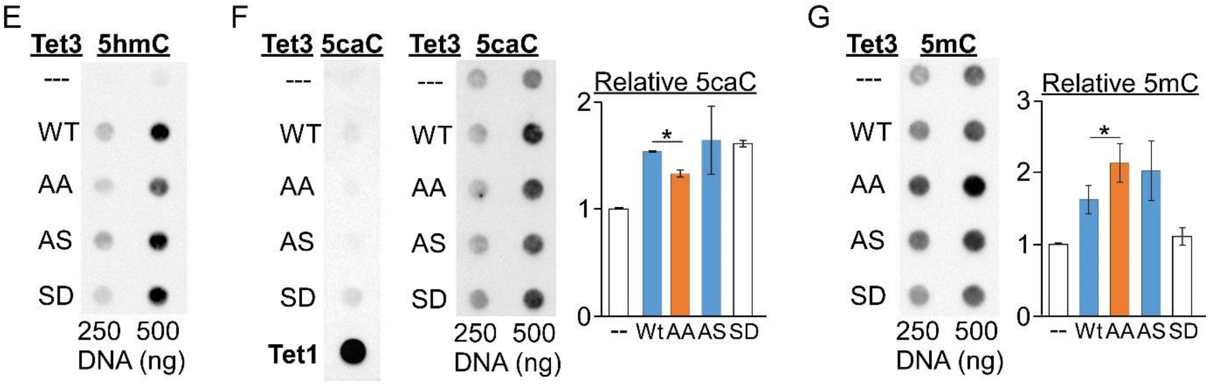

Figure S2

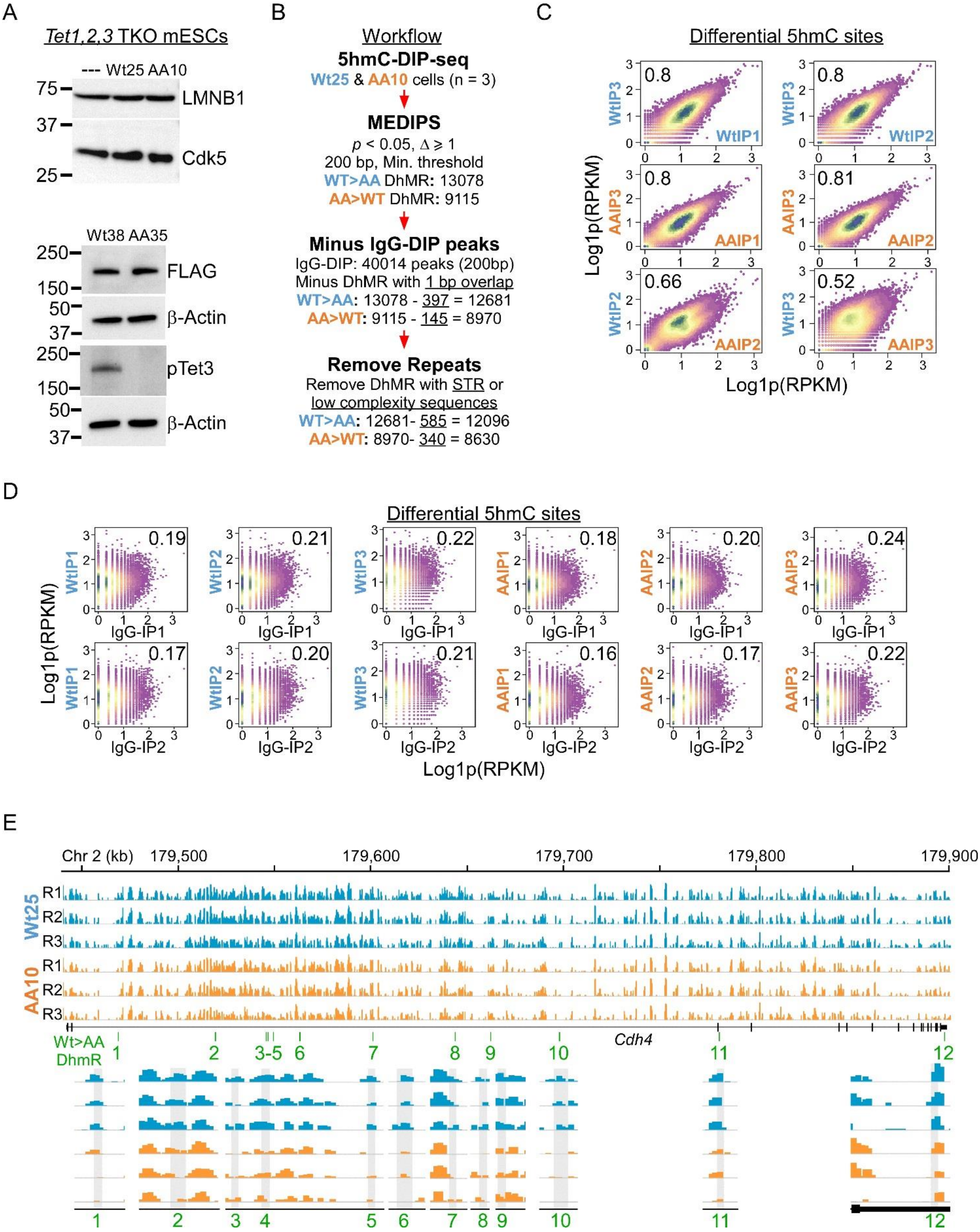

A
Tet1,2,3 TKO mESCs
--- Wt25 AA10
75
37
25
LMNB1
Cdk5
Wt38 AA35
250
150
50
37
FLAG
β-Actin
pTet3
B
Workflow
5hmC-DIP-seq
Wt25 & AA10 cells (n = 3)
MEDIPS
p < 0.05, Δ ⩾ 1
200 bp, Min. threshold
WT>AA DhMR: 13078
AA>WT DhMR: 9115
Minus IgG-DIP peaks
IgG-DIP: 40014 peaks (200bp)
Minus DhMR with 1 bp overlap
WT>AA: 13078 - 397 = 12681
AA>WT: 9115 - 145 = 8970
Remove Repeats
Remove DhMR with STR or
low complexity sequences
WT>AA: 12681- 585 = 12096
AA>WT: 8970- 340 = 8630
C
Differential 5hmC sites
Log1p(RPKM)
WtIP3 WtIP1 0.8
WtIP3 WtIP2 0.8
AAIP3 AAIP1 0.8
AAIP3 AAIP2 0.81
WtIP2 AAIP2 0.66
WtIP3 AAIP3 0.52
D
Differential 5hmC sites
Log1p(RPKM)
WtIP1 IgG-IP1 0.19
WtIP2 IgG-IP1 0.21
WtIP3 IgG-IP1 0.22
AAIP1 IgG-IP1 0.18
AAIP2 IgG-IP1 0.20
AAIP3 IgG-IP1 0.24
WtIP1 IgG-IP2 0.17
WtIP2 IgG-IP2 0.20
WtIP3 IgG-IP2 0.21
AAIP1 IgG-IP2 0.16
AAIP2 IgG-IP2 0.17
AAIP3 IgG-IP2 0.22
E
Chr 2 (kb) 179,500 179,600 179,700 179,800 179,900
Wt25 R1 R2 R3
AA10 R1 R2 R3
Cdh4
Wt>AA DhmR
1 2 3-5 6 7 8 9 10 11 12
1 2 3 4 5 6 7 8 9 10 11 12

Figure S3

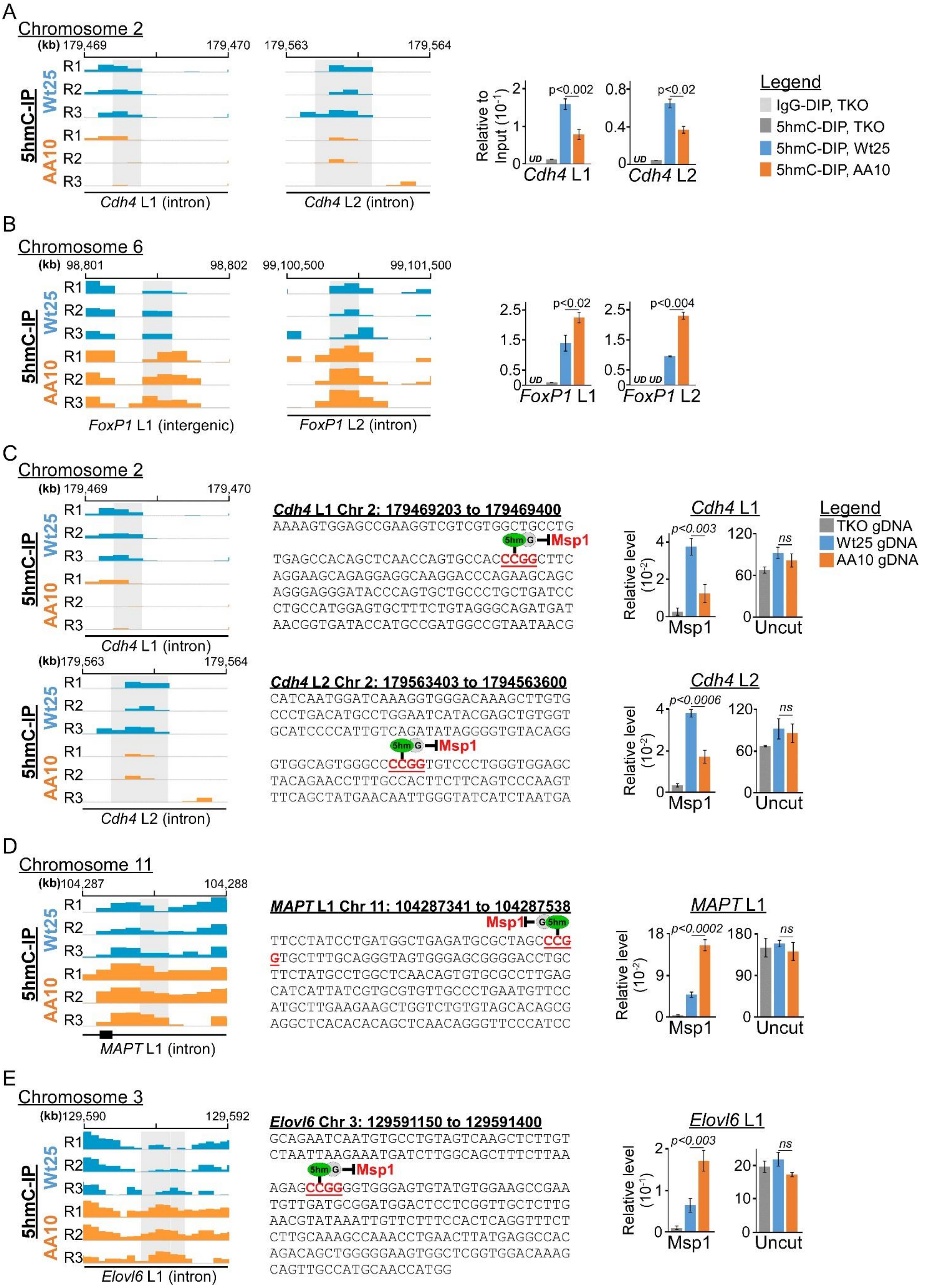
A
Chromosome 2
(kb) 179,469
179,470
179,563
179,564
5hmC-IP
Wt25
AA10
R1
R2
R3
Cdh4 L1 (intron)
Cdh4 L2 (intron)
Relative to Input (10^-1)
p<0.002
p<0.02
UD
Cdh4 L1
Cdh4 L2
Legend
IgG-DIP, TKO
5hmC-DIP, TKO
5hmC-DIP, Wt25
5hmC-DIP, AA10
B
Chromosome 6
(kb) 98,801
98,802
99,100,500
99,101,500
FoxP1 L1 (intergenic)
FoxP1 L2 (intron)
p<0.02
p<0.004
FoxP1 L1
FoxP1 L2
C
Chromosome 2
Cdh4 L1 Chr 2: 179469203 to 179469400
AAAAGTGGAGCCGAAGGTCGTCGTGGCTGCCTG
5hm G Msp1
TGAGCCACAGCTCAACCAGTGCCACCCGGCTTC
AGGAAGCAGAGGAGGCAAGGACCCAGAAGCAGC
AGGGAGGGATACCCAGTGCTGCCCTGCTGATCC
CTGCCATGGAGTGCTTTCTGTAGGGCAGATGAT
AACGGTGATACCATGCCGATGGCCGTAATAACG
Cdh4 L1
Relative level (10^-2)
p<0.003
ns
Msp1
Uncut
Legend
TKO gDNA
Wt25 gDNA
AA10 gDNA
Cdh4 L2 Chr 2: 179563403 to 1794563600
CATCAATGGATCAAAGGTGGGACAAAGCTTGTG
CCCTGACATGCCTGGAATCATACGAGCTGTGGT
GCATCCCCATTGTCAGATATAGGGGTGTACAGG
5hm G Msp1
GTGGCAGTGGGCCCCGGTGTCCCTGGGTGGAGC
TACAGAACCTTTGCCACTTCTTCAGTCCCAAGT
TTCAGCTATGAACAATTGGGTATCATCTAATGA
Cdh4 L2
p<0.0006
D
Chromosome 11
(kb)104,287
104,288
MAPT L1 (intron)
MAPT L1 Chr 11: 104287341 to 104287538
Msp1 G 5hm
TTCCTATCCTGATGGCTGAGATGCGCTAGCCCG
GTGCTTTGCAGGGTAGTGGGAGCGGGGACCTGC
TTCTATGCCTGGCTCAACAGTGTGCGCCTTGAG
CATCATTATCGTGCGTGTTGCCCTGAATGTTCC
ATGCTTGAAGAAGCTGGTCTGTGTAGCACAGCG
AGGCTCACACACAGCTCAACAGGGTTCCCATCC
MAPT L1
p<0.0002
E
Chromosome 3
(kb)129,590
129,592
Elovl6 L1 (intron)
Elovl6 Chr 3: 129591150 to 129591400
GCAGAATCAATGTGCCTGTAGTCAAGCTCTTGT
CTAATTAAGAAATGATCTTGGCAGCTTTCTTAA
5hm G Msp1
AGAGCCGGGGTGGGAGTGTATGTGGAAGCCGAA
TGTTGATGCGGATGGACTCCTCGGTTGCTCTTG
AACGTATAAATTGTTCTTTCCACTCAGGTTTCT
CTTGCAAAGCCAAACCTGAACTTATGAGGCCAC
AGACAGCTGGGGGAAGTGGCTCGGTGGACAAAG
CAGTTGCCATGCAACCATGG
Elovl6 L1
Relative level (10^-1)
p<0.003
ns

Figure S4

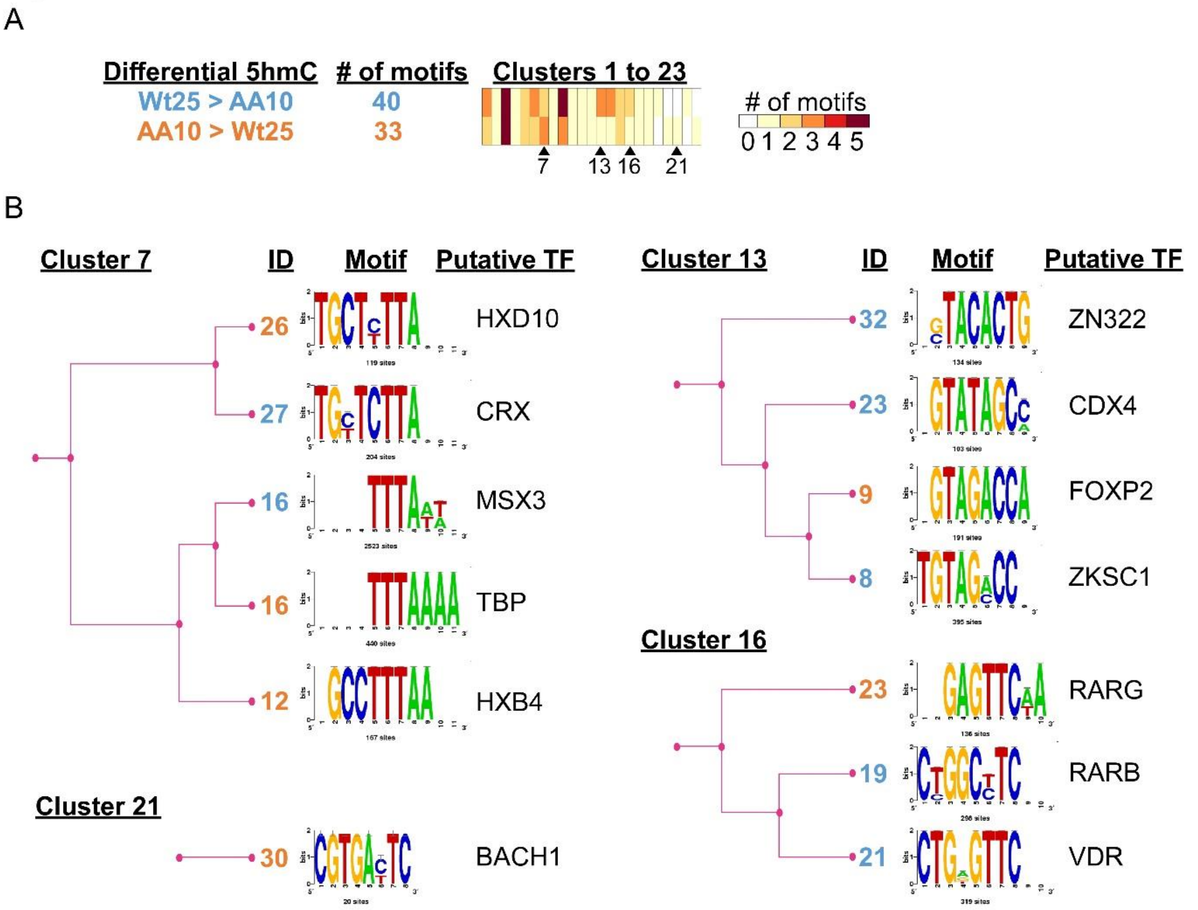
A
Differential 5hmC
# of motifs
Clusters 1 to 23
Wt25 > AA10
40
AA10 > Wt25
33
7
13
16
21
# of motifs
0 1 2 3 4 5
B
Cluster 7
ID
Motif
Putative TF
26
HXD10
27
CRX
16
MSX3
16
TBP
12
HXB4
Cluster 21
30
BACH1
Cluster 13
ID
Motif
Putative TF
32
ZN322
23
CDX4
9
FOXP2
8
ZKSC1
Cluster 16
23
RARG
19
RARB
21
VDR

Figure S5

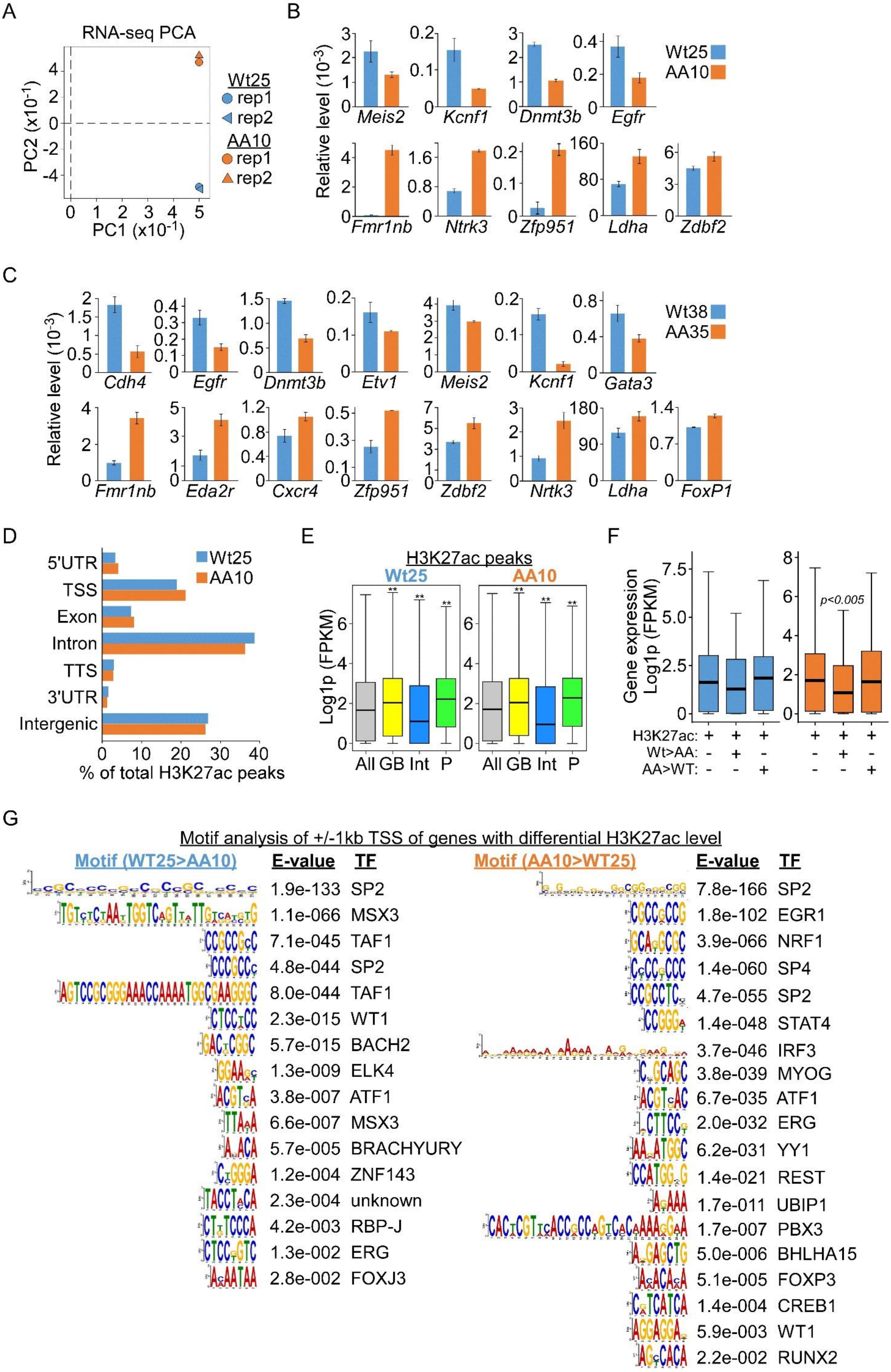

A
RNA-seq PCA
PC2 (x10-1)
PC1 (x10-1)
Wt25
rep1
rep2
AA10
rep1
rep2
B
Relative level (10-3)
Meis2
Kcnf1
Dnmt3b
Egfr
Fmr1nb
Ntrk3
Zfp951
Ldha
Zdbf2
Wt25
AA10
C
Relative level (10-3)
Cdh4
Egfr
Dnmt3b
Etv1
Meis2
Kcnf1
Gata3
Fmr1nb
Eda2r
Cxcr4
Zfp951
Zdbf2
Nrtk3
Ldha
FoxP1
Wt38
AA35
D
5'UTR
TSS
Exon
Intron
TTS
3'UTR
Intergenic
Wt25
AA10
% of total H3K27ac peaks
E
H3K27ac peaks
Wt25
AA10
Log1p (FPKM)
All GB Int P
All GB Int P
F
Gene expression Log1p (FPKM)
p<0.005
H3K27ac:
Wt>AA:
AA>WT:
G
Motif analysis of +/-1kb TSS of genes with differential H3K27ac level
Motif (WT25>AA10) E-value TF
1.9e-133 SP2
1.1e-066 MSX3
7.1e-045 TAF1
4.8e-044 SP2
8.0e-044 TAF1
2.3e-015 WT1
5.7e-015 BACH2
1.3e-009 ELK4
3.8e-007 ATF1
6.6e-007 MSX3
5.7e-005 BRACHYURY
1.2e-004 ZNF143
2.3e-004 unknown
4.2e-003 RBP-J
1.3e-002 ERG
2.8e-002 FOXJ3
Motif (AA10>WT25) E-value TF
7.8e-166 SP2
1.8e-102 EGR1
3.9e-066 NRF1
1.4e-060 SP4
4.7e-055 SP2
1.4e-048 STAT4
3.7e-046 IRF3
3.8e-039 MYOG
6.7e-035 ATF1
2.0e-032 ERG
6.2e-031 YY1
1.4e-021 REST
1.7e-011 UBIP1
1.7e-007 PBX3
5.0e-006 BHLHA15
5.1e-005 FOXP3
1.4e-004 CREB1
5.9e-003 WT1
2.2e-002 RUNX2

Figure S6

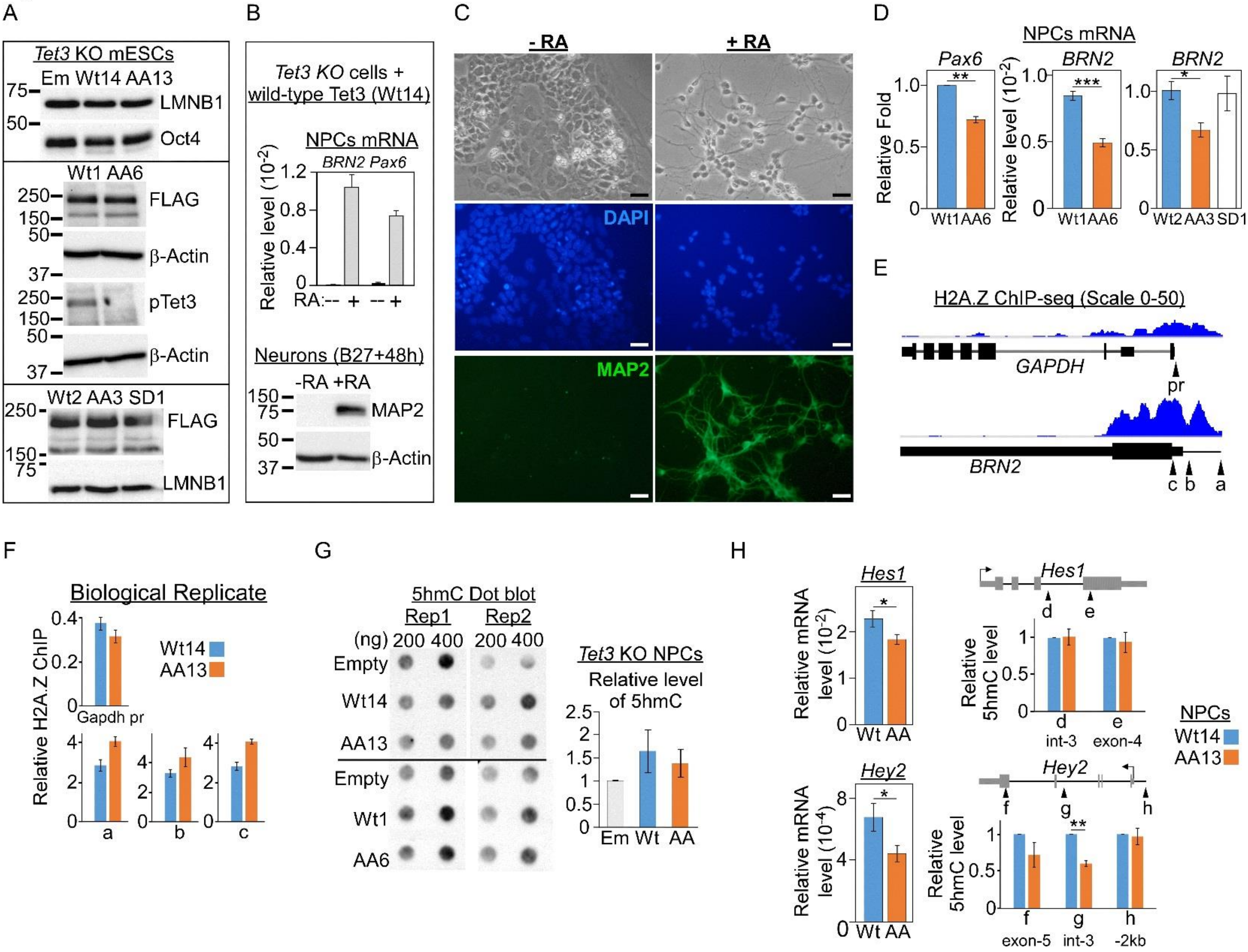

A
Tet3 KO mESCs
Em Wt14 AA13
LMNB1
Oct4
Wt1 AA6
FLAG
β-Actin
pTet3
β-Actin
Wt2 AA3 SD1
FLAG
LMNB1
B
Tet3 KO cells + wild-type Tet3 (Wt14)
NPCs mRNA
BRN2 Pax6
Relative level (10-2)
RA:
Neurons (B27+48h)
-RA +RA
MAP2
β-Actin
C
- RA
+ RA
DAPI
MAP2
D
NPCs mRNA
Pax6
BRN2
BRN2
Relative Fold
Relative level (10-2)
Wt1 AA6
Wt1 AA6
Wt2 AA3 SD1
E
H2A.Z ChIP-seq (Scale 0-50)
GAPDH
pr
BRN2
c b a
F
Biological Replicate
Relative H2A.Z ChIP
Wt14
AA13
Gapdh pr
a
b
c
G
5hmC Dot blot
Rep1
Rep2
(ng) 200 400 200 400
Empty
Wt14
AA13
Empty
Wt1
AA6
Tet3 KO NPCs
Relative level of 5hmC
Em Wt AA
H
Hes1
Relative mRNA level (10-2)
Wt AA
Hes1
d
e
Relative 5hmC level
int-3
exon-4
NPCs
Wt14
AA13
Hey2
Relative mRNA level (10-4)
Wt AA
Hey2
f
g
h
Relative 5hmC level
exon-5
int-3
-2kb

Figure S7

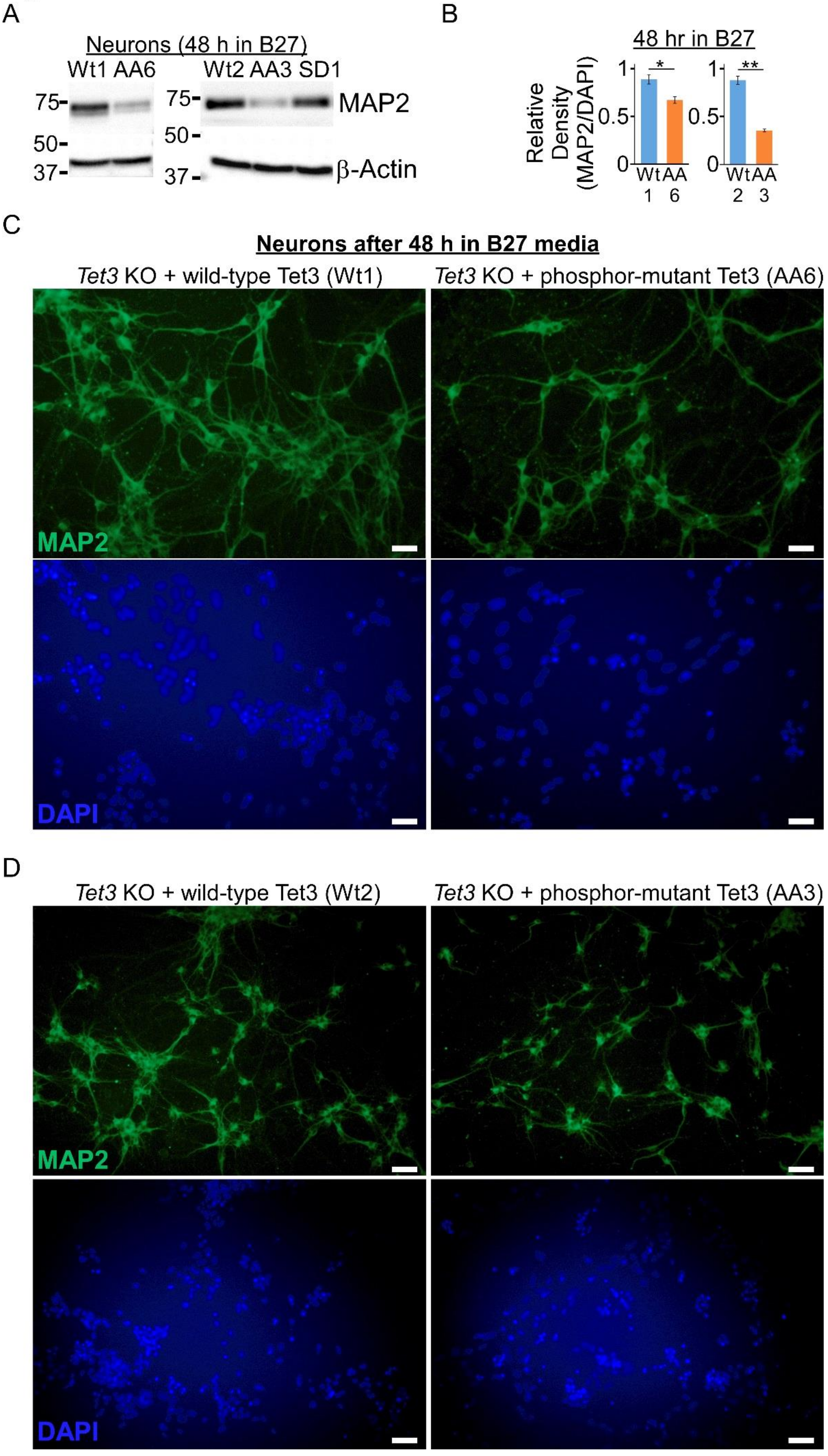
A
Neurons (48 h in B27)
Wt1 AA6
Wt2 AA3 SD1
75
50
37
MAP2
β-Actin
B
48 hr in B27
Relative Density (MAP2/DAPI)
1
0.5
0
*
**
Wt 1
AA 6
Wt 2
AA 3
C
Neurons after 48 h in B27 media
Tet3 KO + wild-type Tet3 (Wt1)
Tet3 KO + phosphor-mutant Tet3 (AA6)
MAP2
DAPI
D
Tet3 KO + wild-type Tet3 (Wt2)
Tet3 KO + phosphor-mutant Tet3 (AA3)
MAP2
DAPI

Figure S8

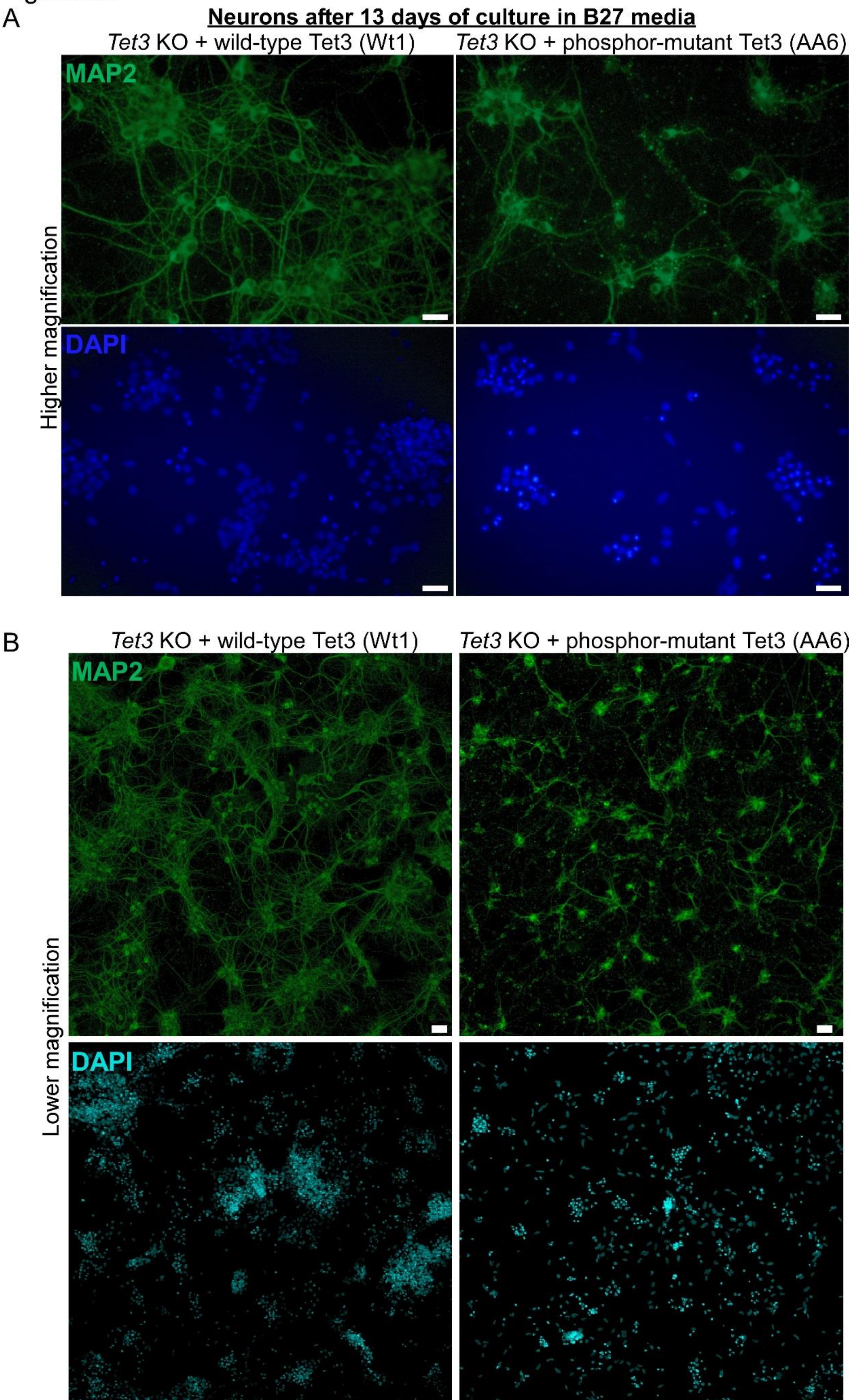
A
Neurons after 13 days of culture in B27 media
Tet3 KO + wild-type Tet3 (Wt1)
Tet3 KO + phosphor-mutant Tet3 (AA6)
MAP2
DAPI
Higher magnification
B
Tet3 KO + wild-type Tet3 (Wt1)
Tet3 KO + phosphor-mutant Tet3 (AA6)
MAP2
DAPI
Lower magnification

Figure S9

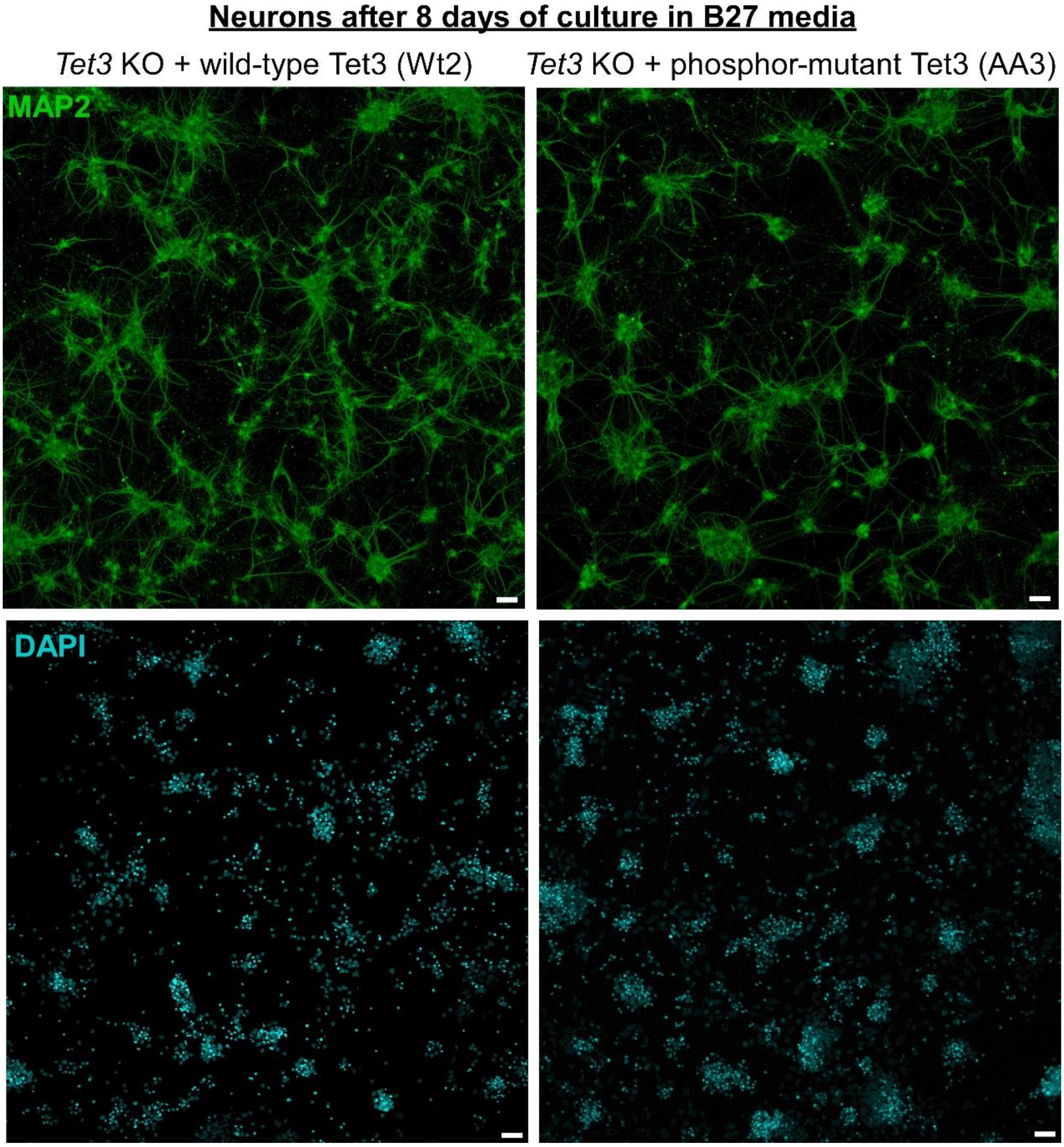
Neurons after 8 days of culture in B27 media
Tet3 KO + wild-type Tet3 (Wt2)
Tet3 KO + phosphor-mutant Tet3 (AA3)
MAP2
DAPI

References

1. Pradeepa, M.M., Grimes, G.R., Kumar, Y., Olley, G., Taylor, G.C., Schneider, R. and Bickmore, W.A. (2016) Histone H3 globular domain acetylation identifies a new class of enhancers. *Nat Genet*, **48**, 681-686.
2. Bustin, S.A., Benes, V., Garson, J.A., Hellemans, J., Huggett, J., Kubista, M., Mueller, R., Nolan, T., Pfaffl, M.W., Shipley, G.L. *et al.* (2009) The MIQE guidelines: minimum information for publication of quantitative real-time PCR experiments. *Clin Chem*, **55**, 611-622.
3. Lienhard, M., Grimm, C., Morkel, M., Herwig, R. and Chavez, L. (2014) MEDIPS: genome-wide differential coverage analysis of sequencing data derived from DNA enrichment experiments. *Bioinformatics*, **30**, 284-286.
4. Hsu, C.C., Zhao, D., Shi, J., Peng, D., Guan, H., Li, Y., Huang, Y., Wen, H., Li, W., Li, H. *et al.* (2018) Gas41 links histone acetylation to H2A.Z deposition and maintenance of embryonic stem cell identity. *Cell Discov*, **4**, 28.
5. Zhang, J., Chen, S., Zhang, D., Shi, Z., Li, H., Zhao, T., Hu, B., Zhou, Q. and Jiao, J. (2016) Tet3-Mediated DNA Demethylation Contributes to the Direct Conversion of Fibroblast to Functional Neuron. *Cell Rep*, **17**, 2326-2339.
