# Supplementary material for: Phosphorylation of Tet3 by cdk5 is critical for robust activation of BRN2 during neuronal differentiation
Source: Nucleic Acids Res. 2019 Dec 6;48(3):1225–38. doi: 10.1093/nar/gkz1144 (PMC7026633; doi:10.1093/nar/gkz1144)
Supplement: gkz1144_Supplemental_Files [file gkz1144_supplemental_files.zip › TableS2_NGS info.pdf]

<u>**Table S2. Summary of next generation sequencing datasets**</u>

A) 5hmC-DNA-IP and H3K27ac ChIP

Sample Name	Sample Description	Paired End (PE) Raw Reads	Aligned PE Reads	PE Reads after removal of duplicates
AA10_5hmCIP_Rep1	5hmC-DNA-IP of AA10 Tet3 expressing mouse ESCs replicate 1	45994734	36021012	31030606
AA10_5hmCIP_Rep2	5hmC-DNA-IP of AA10 Tet3 expressing mouse ESCs replicate 2	47987560	37266993	31585897
AA10_gDNA_5hmC_Rep1	gDNA, input for 5hmCIP of AA10 Tet3 mouse ESCs replicate 1	45739149	38040506	32173894
AA10_gDNA_5hmC_Rep2	gDNA, input for 5hmCIP of AA10 Tet3 mouse ESCs replicate 2	35849067	29934871	24448553
WT25_5hmCIP_Rep1	5hmC-DNA-IP of Wt25 Tet3 expressing mouse ESCs replicate 1	44976850	35023061	30050662
WT25_5hmCIP_Rep2	5hmC-DNA-IP of Wt25 Tet3 expressing mouse ESCs replicate 2	58424245	44879606	38637381
WT25_gDNA_5hmC_Rep1	gDNA, input for 5hmCIP of Wt25 Tet3 mouse ESCs replicate 1	75717559	63850083	52466621
WT25_gDNA_5hmC_Rep2	gDNA, input for 5hmCIP of Wt25 Tet3 mouse ESCs replicate 2	32301439	27069351	23003763
WT25_5hmCIP_Rep3	5hmC-DNA-IP of Wt25 Tet3 expressing mouse ESCs replicate 3	20883520	15113203	12891475
WT25_gDNA_5hmC_Rep3	gDNA, input for 5hmCIP of Wt25 Tet3 mouse ESCs replicate 3	17751878	13912985	12309870
AA10_5hmCIP_Rep3	5hmC-DNA-IP of AA10 Tet3 expressing mouse ESCs replicate 3	39372742	28051863	23417790
AA10_gDNA_5hmC_Rep3	gDNA, input for 5hmCIP of AA10 Tet3 mouse ESCs replicate 3	34941355	27106631	23167869
AA10_K27ac_Rep1	H3K27ac ChIP of AA10 Tet3 expressing mouse ESCs replicate 1	31315237	27228193	23675386
AA10_K27ac_Rep2	H3K27ac ChIP of AA10 Tet3 expressing mouse ESCs replicate 2	33835802	29984464	24656734
AA10_MNase_Rep1	MNase cut, ChIP input of AA10 Tet3 expressing mouse ESCs replicate 1	37351692	31799261	27187897
AA10_MNase_Rep2	MNase cut, ChIP input of AA10 Tet3 expressing mouse ESCs replicate 2	34890434	30221628	24973431
WT25_K27ac_Rep1	H3K27ac ChIP of Wt25 Tet3 expressing mouse ESCs replicate 1	31257013	27958148	23120428
WT25_K27ac_Rep2	H3K27ac ChIP of Wt25 Tet3 expressing mouse ESCs replicate 2	27358760	24170100	20684682
WT25_MNase_Rep1	MNase cut, ChIP input of wt25 Tet3 expressing mouse ESCs replicate 1	36496647	31542220	26044087
WT25_MNase_Rep2	MNase cut, ChIP input of wt25 Tet3 expressing mouse ESCs replicate 2	31031696	26433797	22274912

B) RNA-sequencing

Sample Name	Sample Description	Raw Reads	Aligned Reads
AA10_mRNA_Rep1	RNA-seq of AA10 Tet3 expressing mouse ESCs replicate 1	21,019,994	18605296
AA10_mRNA_Rep2	RNA-seq of AA10 Tet3 expressing mouse ESCs replicate 2	21,021,037	18661990
Wt25_mRNA_Rep1	RNA-seq of Wt25 Tet3 expressing mouse ESCs replicate 1	21,120,732	18758884
Wt25_mRNA_Rep2	RNA-seq of Wt25 Tet3 expressing mouse ESCs replicate 2	21,044,548	18619700
