# Supplementary material for: Phosphorylation of Tet3 by cdk5 is critical for robust activation of BRN2 during neuronal differentiation
Source: Nucleic Acids Res. 2019 Dec 6;48(3):1225–38. doi: 10.1093/nar/gkz1144 (PMC7026633; doi:10.1093/nar/gkz1144)
Supplement: gkz1144_Supplemental_Files [file gkz1144_supplemental_files.zip › TableS7,S8_mESC lines+primers.pdf]

Table S7A. Summary of mouse ESC lines generated

S/N	Genotype of parental ESCs line	Human Tet3 transgene	Mouse ESCs line	Experimental Objective
1	*Tet1,2 and 3 triple knock out (TKO)*	Wild-type	Wt 25	To study how wild-type (Wt) and phosphor-mutant (AA) Tet3 may regulate the genomic distribution of 5hmC and mRNA expression when stably expressed in ESCs that are devoid of endogenous Tet enzymes.
		Phosphor-mutant S1310A, S1379A	AA 10	
2	*Tet1,2 and 3 triple knock out (TKO)*	Wild-type	Wt 38	Second set of independent lines to validate the mRNA expression of key genes identified by RNA-seq of Wt25 and AA10 mouse ESCs lines.
		Phosphor-mutant S1310A, S1379A	AA 35	
3	*Tet 3 Knockout*	Wild-type	Wt 14	To study how wild-type (Wt) and phosphor-mutant (AA) Tet3 may regulate RA-mediated neuronal differentiation.
		Phosphor-mutant S1310A, S1379A	AA 13	
4	*Tet 3 Knockout*	Wild-type	Wt 1	Second set of independent lines to validate the observations made with Wt 14 and AA 13 lines.
		Phosphor-mutant S1310A, S1379A	AA 6	
5	*Tet 3 Knockout*	Wild-type	Wt 2	Third set of independent lines to validate the observations made with the above lines. Wt2 and AA3 lines also act as control to evaluate the function of phosphor-mimic Tet3 (SD1 line).
		Phosphor-mutant S1310A, S1379A	AA 3	
		Phosphor-mimic S1310D	SD 1	

Table S7B. Quantification of neuronal differentiation experiment

Tet3 KO mouse ESCs line	Cell count based on DAPI signal (# of fields taken)		Relative neurons density (MAP2+ area/DAPI)		*p*-value on the individual field	Number of days in B27 media	Biological Replicate	Figure
	WT	AA	WT	AA				
Wt14<br>AA13	1789<br>(6)	2028<br>(6)	0.85	0.64	0.037	12	2	6H<br>6I
Wt1<br>AA6	4279<br>(4)	2413<br>(4)	0.88	0.67	0.006	2	1	S7B<br>S7C
Wt2<br>AA3	2909<br>(4)	4734<br>(4)	0.88	0.35	0.00001	2	1	S7B<br>S7D
Wt1<br>AA6	7200<br>(4)	3931<br>(4)	0.86	0.52	0.002	13	1	6H<br>S8
Wt2<br>AA3	9207<br>(8)	12240<br>(8)	0.83	0.64	0.003	8	1	6H<br>S9

Table S8. Primer sequences

A) Molecular cloning

S/N	Primer Name	Sequence (5'-3')	Use for molecular cloning
1	5'TET3EcoR1	CCGCGAATTCGAGTTCCCTACCTGCGATTGTGTC	Cloning of mouse Tet3 C-term domain into EcoR1 and Not1 sites of GST vector
2	3'TET3Not1	GCGGCCGCCTAGATCCAGCGGCTGTAGGG	
3	S1318A_F	CAGGAGGCCCAAGCATGGCCCCGAAGAGGACTAAC	Mutate mouse Tet3 serine 1318 to alanine.
4	S1318A_R	GTTAGTCCTCTTCGGGGCCATGCTTGGGCCTCCTG	
5	S1387A_F	GCCCCCTGCAAGTTTGGGA	Mutate mouse Tet3 serine 1387 to alanine.
6	S1387A_R	CCATGGCTTGCCTCGAAGC	
7	S1318D_F	ACCCGAAGAGGACTAACAGTGTAGGT	Mutate mouse Tet3 serine 1318 to aspartic acid.
8	S1318D_R	CCATGCTTGGGCCTCCTGAGTAC	
9	HumS1310A_F	CCAAGCATGGCCCCCAAGAGGACTAACGG	Mutate human Tet3 serine 1310 to alanine.
10	HumS1310A_R	TCTTGGGGGCCATGCTTGGGCCTCCTG	
11	HumS1379A_F	AAACCGTGGGCCCCCTGCAAGTTTGGGAACAGC	Mutate human Tet3 serine 1379 to alanine.
12	HumS1379A_R	CTTGCAGGGGGCCCACGGTTTGCCTCGC	
13	HumS1310D_F	CCAAGCATGGACCCCAAGAGGACTAACGG	Mutate human Tet3 serine 1310 to aspartic acid.
14	HumS1310D_R	TCTTGGGGTCCATGCTTGGGCCTCCTG	
15	5'TET3Xba1	ATCTAGAATGGACTCAGGGCCAGTGTACC	Cloning of human full lengthTet3 into the Xba1 and PspX1 sites of pLV-EFα-IRES-puro vector.
16	3'TET3Sal1	TGTCGACCTAGATCCAGCGGCTGTAGG	

B) qPCR Validation of mRNA expression

S/N	Primer Name	Sequence (5'-3')	Position
1	Etv1	F: TTAAGTGCAGGCGTCTTCTTC<br>R: GGAGGCCATGAAAAGCCAAA	Exons 1 & 2
2	Meis2	F: TGTCCTCGTTGAAGGAGTCAG<br>R: GCTCTGGTTTTTGAGAAGTGC	Exons 3 & 4
3	Kcnf1	F: AACCTTTCGGCAAATCTCCT<br>R: CCAGCCCTCTCACTTAGGAA	Exons 1 & 2
4	CxCr4	F: GACTGGCATAGTCGGCAATG<br>R: AGAAGGGGAGTGTGATGACAAA	Exons 2 & 1
5	Zdbf2	F: TTTGAGTGTTCGAGGCCAGT<br>R: TTAGTATACTGCACACGGCAAT	Exons 3 & 4
6	Ntrk3	F: CTCAATGCTGTGGACATGGA<br>R: GGCTGGATGTTCCGGAGT	
7	Cdh4	F: CAGGCCACTGACATGGAAGG<br>R: ATGATTCGGTAGACGGCGTTC	Exons 8 & 9
8	Egfr	F: CTCGTGGAACCTCTCACACC<br>R: CCCGAACCCAGAACTTTGAT	Exons 18 & 19
9	Dnmt3b	F: CTAGCAGGAGCCACCCAAGT<br>R: AAACTCACCCGTTGCAATTC	Exons 15 & 16
10	Fmr1nb	F: TCTAAGAGCCGTGATTATCGTGG<br>R: TGAGGGCAAGAGTATCGTCTC	Exons 1 & 2
11	Eda2r	F: ATGAGTACCGGGACCAATGG<br>R: GAGGGCAGACTATGCAGTGTG	Exons 3 & 2
12	Zfp951	F: TTGTGCTTCGACCTCAACTG<br>R: TGTGCAGGTCCATGAAACAT	Exons 1 & 2
13	NeuroD1	F: GAACCTTTTAACAACAGGAAGTGG<br>R: GTCTCTTGGGCTTTTGATCC	Exons 2 & 1
14	Pax6	F: CCGCCCTCACCAACACGTACAGT<br>R: TTGCATGTGCGGAGGGGTGTAG	SG Jin et al; 2016
15	Hey2	F: GGTAGTTGTCGGTGAATTGGAC<br>R: AAGCGCCCTTGTGAGGAAAC	Primer bank ID 7305159a1

16	BRN2	F: GCAGCGTCTAACCACTACAGC<br>R: GCGGTGATCCACTGGTGAG	Primer bank ID 6679423a1
17	Hes1	F: CCAGCCAGTGTCAACACGA<br>R: AATGCCGGGAGCTATCTTTCT	Primer bank ID 6680205a1
18	GAPDH	F: AGGTCGGTGTGAACGGATTTG<br>R: GGGGTCGTTGATGGCAACA	Primer bank ID 126012538c1
19	FoxP1	F: AAGTGTTTTGTGCGAGTAGAGAA<br>R: GGGAAGGGTTACCACTGATCTT	Primer bank ID 16716509a1
20	Ldha	F: TGTCTCCAGCAAAGACTACTGT<br>R: GACTGTACTTGACAATGTTGGGA	Primer bank ID 6754524a1
21	Gata3	F: AAGCTCAGTATCCGCTGACG<br>R: GTTTCCGTAGTAGGACGGGAC	Primer bank ID 160948602c1

C) 5hmC-DNA-IP and H2A.Z ChIP-qPCR

S/N	Primer Name	Sequence (5'-3')	Location
1	BRN2 a	F: CGAGTCAGCCAGCAGGACTA<br>R: GGACACCTGTGCGCTGTAAT	-634 bp to -934 bp upstream of TSS
2	BRN2 b	F: CGTAAATCAAAGGGCGCAGA<br>R: TCCTCCTCTCCCTCCTCTCG	-14bp to -187 bp upstream of TSS
3	BRN2 c	F: GCGGCAGCAGCAGTAATAGC<br>R: AGCCGCGCATTTGACAGTTA	+22 bp to +199 bp from TSS
4	Hes1 intron3	F: GTTCCCACGGTCTGGGTCTT<br>R: TCACCGGCTTCTACCACAGC	chr16:30066453-30066802 mm9
5	Hes1 exon4	F: CACCAGCAACAGTGGGACCT<br>R: TACGGGTAGCAGTGGCCTGA	chr16:30067227-30067576 mm9
6	Hey2 exon5	F: AACTTGGGTCTTGGTGGCTTCT<br>R: AGCTGCTGAAGAACGGACACTC	chr10:30558605-30558954 mm9
7	Hey2 intron3	F: GATGGCATCCGAAGAGCAGA<br>R: AGCCCAATGGACTCCACACA	chr10:30553810-30554159 mm9
8	Hey2 -2kb TSS	F: TAGCGCTGGGTGGGTTAGTG<br>R: TGGAGGGTCTCGGTGATTGA	chr10:30563739-30564088 mm9
9	GAPDH_PR	F: GCCTCTGAGCCTCCTCCAAT<br>R: TGCAGCCTGGAAACCTGATAAT	-50 bp to +100 bp from TSS

D) 5hmC-IP RT-PCR and *MspI* validation

S/N	Primer Name	Sequence (5'-3')	Location
1	Cdh4 L1	F1: CAAAAGTGGAGCCGAAGGT<br>R1: GCCCTACAGAAAGCACTCCA	Chr 2:179469150-179469450
2	Cdh4 L2	F7: TGCATCCCCATTGTCAGATA<br>R7: GCAGACAATTCTGCAATCCA	Chr 2:179563400-179563700
3	Cdh4 no CCGG control	F: CATCTGTCTGCGTCCCTGTG<br>R: AGGTTCCCTGGGGCTGAA	Chr 2:179644000-179644202
4	FoxP1 L1	F2: CAGTTGGATGAAATTTTTAAGCTGTC<br>R2: AAAGCCCAGCCCTTCAACAT	Chr 6:98801350-98801650
5	FoxP1 L2	F3: CCATTAAAGAGCTACCATTTTTAGCA<br>R3: GGTGACATTCAAGTCCAGCAGA	Chr 6:99099750-99100000
6	MAPT MspI	F: TTCCTATCCTGATGGCTGAGA<br>R: GTTGAGCCAGGCATAGAAGC	Chr 11: 104287341-104287424
7	MAPT no CCGG	F: TACCTTCCATGACCCCCTCA<br>R: TGAGCGGGAGACTTCTGACC	Chr 11:104287500-104287700
8	Elovl6 MspI	F: GCAGAATCAATGTGCCTGTAGTC<br>R: AAGAGCAACCGAGGAGTCCA	Chr 3:129591150-129591400
9	Elovl6 no CCGG	F: CTAGCCAGCTGCCATTAGGC<br>R: TGCGAACAAATGACACACGA	Chr 3: 129590802-129591000
